# JMJD1A is a signal-sensing scaffold that regulates acute chromatin dynamics via SWI/SNF association for thermogenesis

**DOI:** 10.1038/ncomms8052

**Published:** 2015-05-07

**Authors:** Yohei Abe, Royhan Rozqie, Yoshihiro Matsumura, Takeshi Kawamura, Ryo Nakaki, Yuya Tsurutani, Kyoko Tanimura-Inagaki, Akira Shiono, Kenta Magoori, Kanako Nakamura, Shotaro Ogi, Shingo Kajimura, Hiroshi Kimura, Toshiya Tanaka, Kiyoko Fukami, Timothy F. Osborne, Tatsuhiko Kodama, Hiroyuki Aburatani, Takeshi Inagaki, Juro Sakai

**Affiliations:** 1 Division of Metabolic Medicine, Research Center for Advanced Science and Technology (RCAST), The University of Tokyo, Tokyo 153-8904, Japan; 2 Department of Cardiology and Vascular Medicine, Faculty of Medicine, Gadjah Mada University, Yogyakarta 55281, Indonesia; 3 Laboratory for Systems Biology and Medicine, Research Center for Advanced Science and Technology (RCAST), The University of Tokyo, Tokyo 153-8904, Japan; 4 The Translational Systems Biology and Medicine Initiative, Center for Disease Biology and Integrative Medicine, Faculty of Medicine, University of Tokyo, Tokyo 113-8655, Japan; 5 Genome Science Division, Research Center for Advanced Science and Technology (RCAST), The University of Tokyo, Tokyo 153-8904, Japan; 6 UCSF Diabetes Center, Department of Cell and Tissue Biology, University of California, San Francisco, San Francisco, California 94143-0669, USA; 7 Graduate School of Frontier Biosciences, Osaka University, Suita, Osaka 565-0871, Japan; 8 Laboratory of Genome and Biosignals, Tokyo University of Pharmacy and Life Science, Tokyo 192-0392, Japan; 9 Metabolic Disease Program, Sanford-Burnham Medical Research Institute, Orlando, Florida 32827, USA

## Abstract

Histone 3 lysine 9 (H3K9) demethylase JMJD1A regulates β-adrenergic-induced systemic metabolism and body weight control. Here we show that JMJD1A is phosphorylated at S265 by protein kinase A (PKA), and this is pivotal to activate the β1-adrenergic receptor gene (*Adrb1*) and downstream targets including *Ucp1* in brown adipocytes (BATs). Phosphorylation of JMJD1A by PKA increases its interaction with the SWI/SNF nucleosome remodelling complex and DNA-bound PPARγ. This complex confers β-adrenergic-induced rapid JMJD1A recruitment to target sites and facilitates long-range chromatin interactions and target gene activation. This rapid gene induction is dependent on S265 phosphorylation but not on demethylation activity. Our results show that JMJD1A has two important roles in regulating hormone-stimulated chromatin dynamics that modulate thermogenesis in BATs. In one role, JMJD1A is recruited to target sites and functions as a cAMP-responsive scaffold that facilitates long-range chromatin interactions, and in the second role, JMJD1A demethylates H3K9 di-methylation.

## Abstract (web summary)

Demethylase JMJD1A activates thermogenesis-related target genes in response to β-adrenergic hormones. Here, the authors show that phosphorylation of JMJD1A at S265 increases its interaction with the SWI/SNF chromatin remodeler, leading to long-range chromatin interactions and target gene activation.

## Graphical abstract

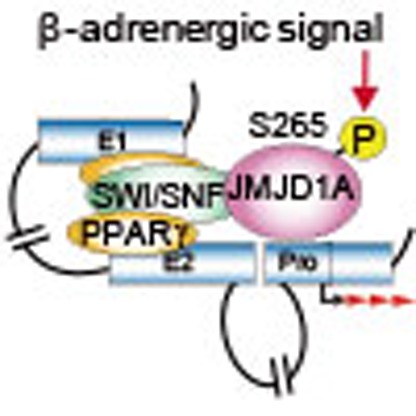

The coupling of environmental cues with the regulation of gene transcription is a very important step in all cellular adaptive responses. For example, catecholamines released from the sympathetic nervous system in response to cold exposure bind to β-adrenergic receptors (β-ARs), and trigger the downstream cyclic AMP (cAMP) signalling cascade. The cAMP-dependent protein kinase A (PKA) phosphorylates a variety of downstream target substrates (for example, cAMP-responsive element binding protein (reviewed in ref. 1)) to transcriptionally upregulate energy expenditure genes^2^,^3^. Recent evidence suggests that in addition to transcription factors (TFs), histone modification enzymes such as histone methyltransferases and demethylases play essential roles in gene transcription and adaptive responses^4^.

JMJD1A (Jumonji domain containing 1A, also referred to as KDM3A or JHDM2A), a member of the Jumonji C-domain containing histone demethylase family, catalyses removal of H3K9 mono- and di-methylation (H3K9me1 and H3K9me2; ref. 5) and functions as a co-activator for androgen receptor, as well as a crucial regulator in spermatogenesis, germ cell development, sex determination, tumorigenesis and hypoxia-inducing factor-1α-mediated gene transcription^5^,^6^,^7^,^8^,^9^,^10^,^11^,^12^. Although JMJD1A regulates a wide array of appropriate gene targets in different settings, this enzyme lacks intrinsic DNA sequence specificity. Therefore, how JMJD1A is targeted to specific genes in response to given environmental stimuli was largely unknown and of current interest.

We and another group reported that JMJD1A deficiency results in obesity with defects in brown adipose tissue functions that lead to cold intolerance and decreased oxygen consumption^13^,^14^. At the molecular level, β-adrenergic stimulation induces binding of JMJD1A to the uncoupling protein 1 gene (*Ucp1*) whose product is pivotal for heat generation. This β-adrenergic-induced binding of JMJD1A to a proximal *Ucp1* enhancer region is a critical step for subsequent gene activation; however, how β-adrenergic stimulation triggers JMJD1A recruitment to *Ucp1* and other genes involved in energy expenditure in BATs has remained elusive.

The chromatin remodelling SWI/SNF (SWItch/Sucrose NonFermentable) complex couples the perturbation of histone–DNA contacts with promoter access by TFs to their cognate DNA elements^15^. SWI/SNF reportedly has a potential role in long-range genomic interactions (reviewed in ref. 16); however, whether rapid environmental changes that alter cell activity in response to hormone signalling (that is, catecholamines) contribute to higher-order chromatin conformational changes and whether SWI/SNF is involved in such rapid action have not been reported.

Post-translational modifications allow proteins to play multiple roles in different physiological contexts. Thus, histone modification enzymes are feasible targets of post-translational modifications that enable cells to adopt various environmental changes. In the current study, we show that JMJD1A is phosphorylated at serine 265 by PKA downstream from β-adrenergic stimulation. This modification facilitates JMJD1A interaction with SWI/SNF and DNA-bound peroxisome proliferator-activated receptor-γ (PPARγ). This phosphorylation switch in JMJD1A is independent of its demethylase activity, suggesting that it plays a scaffolding role to mediate long-range chromatin interactions that position distal enhancers in close proximity to target gene promoters for key thermogenic genes.

## Results

### β-Adrenergic-dependent genomic localization of JMJD1A

**Figure 1:**
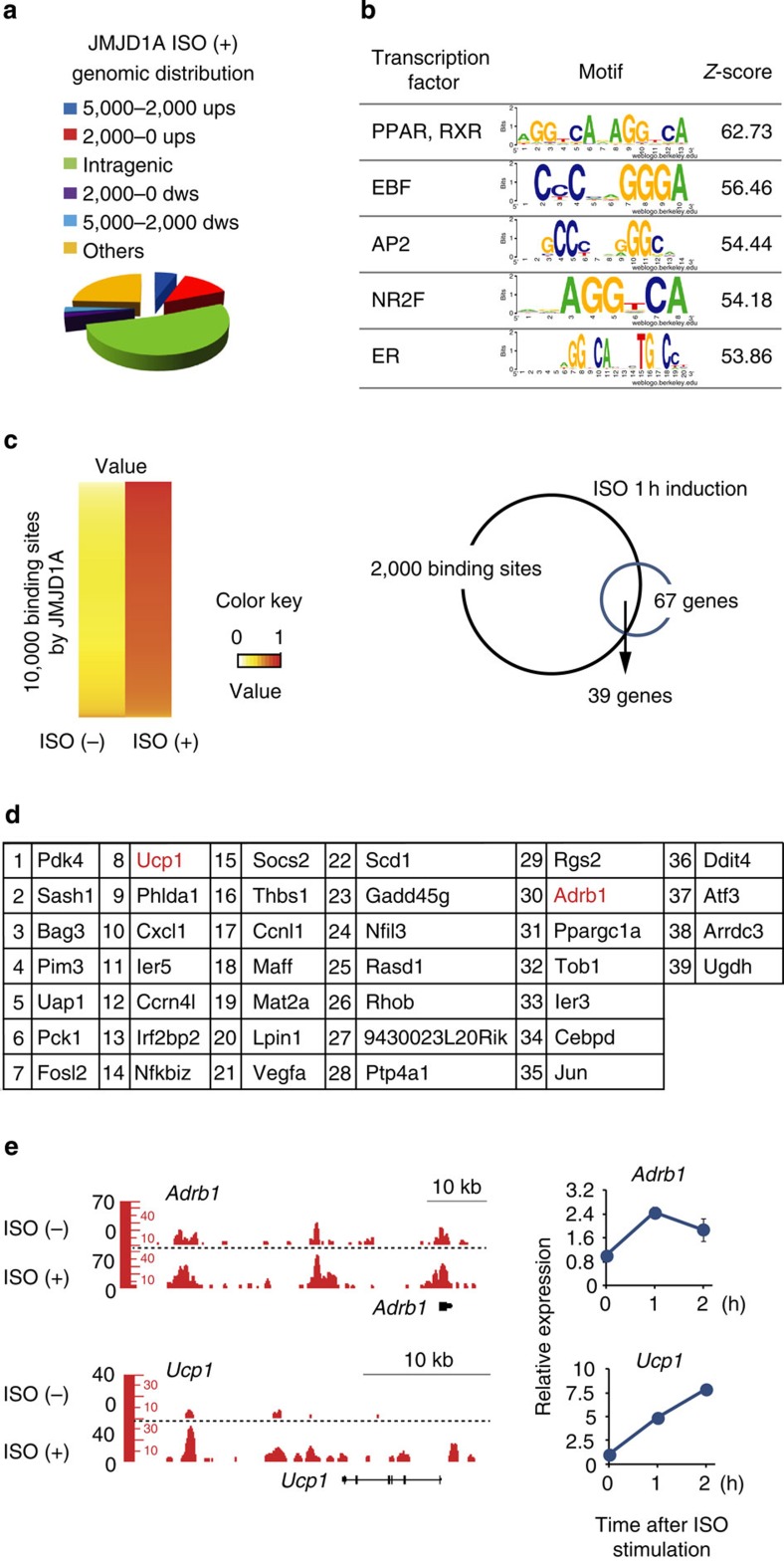
β-Adrenergic-dependent genomic localization of JMJD1A. (**a**) Genome-wide distribution of JMJD1A binding sites in ISO (1 μM for 2 h) treated iBATs. Ups, upstream; dws, downstream; ISO, isoproterenol. (**b**) Table depicting TF binding motifs enriched at constituent enhancers within JMJD1A binding regions relative to genomic background and associated *Z*-scores. (**c**,**d**) JMJD1A ChIP-seq and transcriptional microarray analysis performed in iBATs at day 8 of differentiation (day 8). Heatmap represents top 10,000 high SICER scored JMJD1A binding sites under ISO-plus condition (1 μM for 2 h). Colour intensity represents *Z*-score of JMJD1A binding sites under ISO-minus versus those under ISO-plus condition. The higher the (red/yellow) contrast it becomes, the higher ISO-induced JMJD1A recruitment to the given binding sites relative to ISO-minus. For reference, a colour intensity scale is included (**c**, left panel). Venn diagram showing the top 2,000 sites annotated ISO-induced JMJD1A binding and the number of ISO-induced genes >2^0.8^-fold by ISO (1 μM for 1 h; **c**, right panel). The overlapping genes were listed in **d**. (**e**) Genome browser shots showing the ISO-induced JMJD1A recruitments on selected genomic regions analysed by ChIP-seq in iBATs (day 8) treated with ISO (1 μM) or vehicle for 2 h (left panel). mRNA levels of *Adrb1* and *Ucp1* in iBATs (day 8) after ISO (1 μM) treatment at the indicated time points. Data were presented as fold change relative to 0 h (mean±s.e.m.) of three technical replicates (error bars are too tiny to see; right panel).

To analyse the JMJD1A-dependent transcriptional programme during β-adrenergic stimulation, we combined chromatin immunoprecipitation (ChIP)-seq and global gene expression analyses. Immortalized pre-BATs (namely, pre-iBATs) were differentiated and ChIP-seq was conducted using a newly generated monoclonal anti-mouse JMJD1A antibody at 0 time and 2 h following treatment with the β-AR pan-agonist isoproterenol (ISO). ChIP-seq peak calling by SICER identified 27,397 genomic regions as significant binding sites of JMJD1A in ISO-treated iBATs. JMJD1A localized on proximal promoters (∼13%), intragenic (∼52%) and intergenic regions (∼24%; Fig. 1a). The sequencing tag density was concentrated within proximal regions of transcription start sites (TSSs; Supplementary Fig. 1a). JMJD1A peaks were significantly enriched for clusters of sequence motifs bound by PPAR with the highest *Z*-score of 62.7 in the JMJD1A-associated regions with the 10,000 highest SICER scores (Fig. 1b).

Global gene expression analysis in iBATs following the same ISO stimulation treatment identified 67 genes that were induced by 2^0.8^-fold after 1 h ISO treatment (that is, ISO dependent). Compared with the top 2,000 ISO-induced JMJD1A binding sites, which were annotated to 1,551 proximal genes, 39 out of 67 ISO-induced genes were found to be occupied by JMJD1A (Fig. 1c,d). These genes included *Ucp1*, *Ppargc1a*, *Pdk4*, *Pck1* and *Adrb1*, which are all important metabolic factors induced following β-adrenergic stimulation^17^,^18^ (Fig. 1d,e and Supplementary Fig. 1b). The sequence tag-mapping in Fig. 1e and Supplementary Fig. 1b revealed that JMJD1A was significantly enriched at the genomic loci for these key metabolic genes.

### JMJD1A is phosphorylated by PKA at serine 265

**Figure 2:**
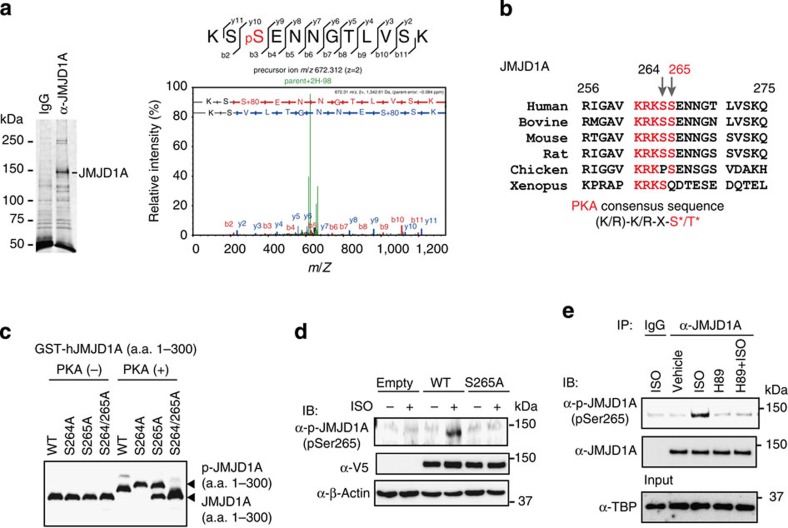
JMJD1A is phosphorylated at serine 265 by PKA. (**a**) Post-translational modifications of JMJD1A identified by mass spectrometry. JMJD1A protein in ISO-treated HeLa cells were immunoprecipitated with anti-hJMJD1A antibody (IgG-F0026), separated by SDS–PAGE gel, stained with SYPRO Ruby and then subjected to in-gel digestion for mass spectrometry (left panel). MS/MS spectrum of the P-JMJD1A fragment from K263 to K274, *m/z*=672.312 (*Z*=2) is shown in the right panel. (**b**) The PKA consensus site is conserved in various species. (**c**) *In vitro* PKA kinase assay. WT, S264, S265A or S264A/S265A mutated JMJD1A (a.a. 1–300) recombinant GST-fusion proteins were PKA-treated and subjected to Phos-tag SDS–PAGE followed by immunoblot (IB) analysis with anti-GST antibody. (**d**) ISO-induced JMJD1A phosphorylation at S265. Whole-cell lysates from WT- or S265A-hJMJD1A expressing iBAT^sh^s (day 8) treated with ISO (10 μM for 1 h) were subjected to immunoprecipitation (IP) using anti-mJMJD1A (IgG-F0618) followed by IB analysis with anti-P-S265-JMJD1A. (**e**) IB analysis showing PKA-mediated phosphorylation of native JMJD1A at S265. iBATs (day 8) were pretreated with PKA inhibitor H89 (20 μM) for 20 min and then treated with ISO (10 μM) for 1 h. Whole-cell lysates were subjected to IP using anti-mJMJD1A (IgG-F0618) and IB analysis with anti-P-S265-JMJD1A. Uncropped images of the blots (**c**–**e**) are shown in Supplementary Fig. 11.

Whether JMJD1A might be phosphorylated following ISO treatment was evaluated by using liquid chromatography-tandem mass spectrometry (LC/MS/MS) analysis. The immunoprecipitated JMJD1A from HeLa cells treated with ISO for 1 h was excised from an SDS–PAGE gel and subjected to LC/MS/MS (Fig. 2a). This analysis revealed that JMJD1A was phosphorylated at two consecutive residues on amino acids S264 and S265. These residues were found as part of a putative consensus sequence for PKA phosphorylation (Fig. 2b). *In vitro* phosphorylation assays demonstrated that PKA phosphorylated recombinant human JMJD1A (hJMJD1A; amino acids (a.a.) 1–300) at S265 (Fig. 2c). Approximately 50% of the S265A mutant protein was not phosphorylated and the S264/265A double mutant was no longer phosphorylated by PKA, while PKA phosphorylation was retained in S264A mutant (Fig. 2c). These data suggest that S265 is likely the major PKA phosphorylation site.

Immunoblot analysis with a newly generated phospho-specific antibody against phospho-S265-JMJD1A detected WT-JMJD1A transiently expressed in iBATs cultured under ISO-plus conditions; however, this antibody failed to detect the S265A-JMJD1A mutant (Fig. 2d). Immunoprecipitated JMJD1A from lysates of iBATs pretreated with ISO was detected by anti-P-S265-JMJD1A antibody but the antibody failed to recognize JMJD1A in cells pretreated with the PKA inhibitor H89 (Fig. 2e).

### Phospho-S265 is dispensable for enzymatic activity *in vitro*

To examine whether S265 phosphorylation might alter JMJD1A demethylase activity, we performed an *in vitro* demethylation assay. H3K9me2 demethylation activity of JMJD1A did not differ between phosphorylated and non-phosphorylated full-length recombinant JMJD1A (Supplementary Fig. 2a). In addition, mutation of S265 to alanine (S265A) did not alter the substrate specificity or demethylase activity (Supplementary Fig. 2b). Both wild-type (WT) and S265A recombinant full-length JMJD1A catalysed the removal of mono- and di-methylation of H3K9 but not tri-methylation (Supplementary Fig. 2c). Immunohistochemistry and subcellular fractionation showed that both WT and S265A mutant JMJD1A predominantly localized in the nucleus with lesser staining in the cytosol of iBATs (Supplementary Fig. 2d,e).

### Gene activation by JMJD1A requires phosphorylation at S265

**Figure 3:**
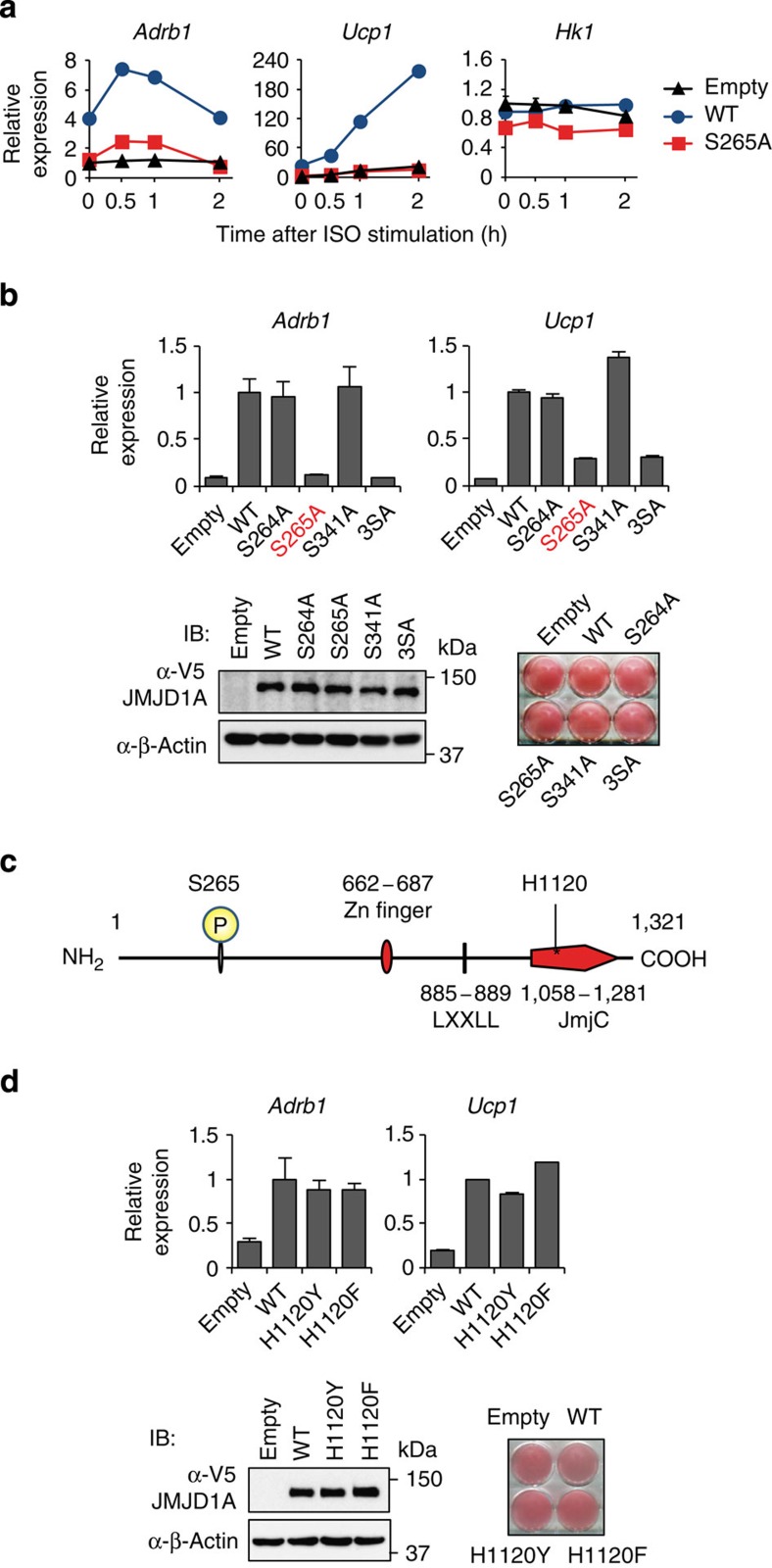
Phosphorylation of JMJD1A at S265 is crucial for β-adrenergic-induced gene transcriptions. (**a**) ISO-induced *Adrb1* and *Ucp1* mRNA levels in iBAT^sh^s stably expressing WT- or S265A-hJMJD1A or empty vector were measured by RT-qPCR. The mRNA values are depicted relative to mRNA in iBAT^sh^s transduced with empty vector on day 8 of differentiation before ISO treatment (0 h), which are arbitrarily defined as 1. (**b**) *Adrb1* and *Ucp1* mRNA levels in WT or serine to alanine mutants (S264A, S265A, S341A or, 3SA) JMJD1A expressing iBAT^sh^s measured by RT-qPCR after 1 h ISO treatment (top panel). 3SA represents all three mutations of S264A, S265A and S341A. Data were presented as fold change relative to WT-hJMJD1A-iBAT^sh^s after normalized to cyclophilin. Immunoblot (IB) analysis for WT and various mutant JMJD1A proteins and Oil Red O (ORO) staining (bottom panel). (**c**) Schematic representation of the domain architecture of hJMJD1A. Phosphorylation site at S265 and Fe(II) binding site at H1120 are shown. (**d**) Comparable ISO-induced gene expressions of *Adrb1* and *Ucp1* in WT and demethylase dead JMJD1A mutants expressing iBAT^sh^s. RT-qPCR was performed to quantify mRNA levels of *Adrb1* and *Ucp1* genes in WT-, H1120Y- or H1120F-hJMJD1A- iBAT^sh^s treated with ISO for 1 h (top panel). Data were presented as fold change relative to WT-hJMJD1A-iBAT^sh^s. IB analysis and ORO in the indicated iBATs (bottom panel). Data are presented as mean±s.e.m. of three technical replicates (**a**,**b**,**d**) (error bars are too tiny to see in some figures). Uncropped images of the blots (**b**,**d**) are shown in Supplementary Fig. 11.

To determine the impact of S265 phosphorylation on gene transcription, we retrovirally transduced either a V5-tagged WT-hJMJD1A, a mutant S265A human version of JMJD1A (S265A-hJMJD1A) or the control *Zeo*^*r*^-empty vector into iBATs where we also knocked down expression of the endogenous mouse JMJD1A by short hairpin RNA (shRNA; iBAT^sh^s). Human *Jmjd1a* is resistant to this shRNA designed against mouse *Jmjd1a* and the retroviral vector is driven by a very weak long terminal repeat promoter. Immunoblot analysis showed that protein level of V5-hJMJD1A was equivalent to that of native JMJD1A (Supplementary Fig. 3a). Reverse transcription–quantitative real-time PCR (qPCR) analyses revealed that transduction of WT-hJMJD1A into iBAT^sh^s upregulates ISO-induced expression of key metabolic genes such as *Ucp1* and *Adrb1*; however, S265A-hJMJD1A was incapable of stimulating gene expression even though it was expressed at levels similar to the WT protein (Fig. 3a and Supplementary Fig. 3a). In contrast, expression of a white adipocyte marker gene, *Hk1*, did not differ significantly between WT- and S265A-hJMJD1A-iBAT^sh^s (Fig. 3a). The position of S265 was highly specific: because substitution of other serine residues nearby such as S264 or S341 to alanine (S264A and S341A, respectively) did not perturb ISO-induced *Adrb1* and *Ucp1* expression, meanwhile S265A and S264/265/341A (3SA mutant) reduced expression (Fig. 3b). It is also notable that mutation of key residues required for demethylase activity, H1120Y or H1120F, (ref. 5) did not affect the ISO-induced expression of *Adrb1* and *Ucp1* (Fig. 3c,d). ChIP–qPCR analysis revealed that ISO treatment of iBATs did not affect H3K9me2 levels at *Adrb1* gene locus (Supplementary Fig. 3b). These results indicate that phospho-S265-JMJD1A (P-JMJD1A)-induced gene transcription appears to be independent of H3K9 demethylase activity at *Adrb1* locus.

### PKA-induced P-JMJD1A–SWI/SNF-PPARγ protein complex

**Figure 4:**
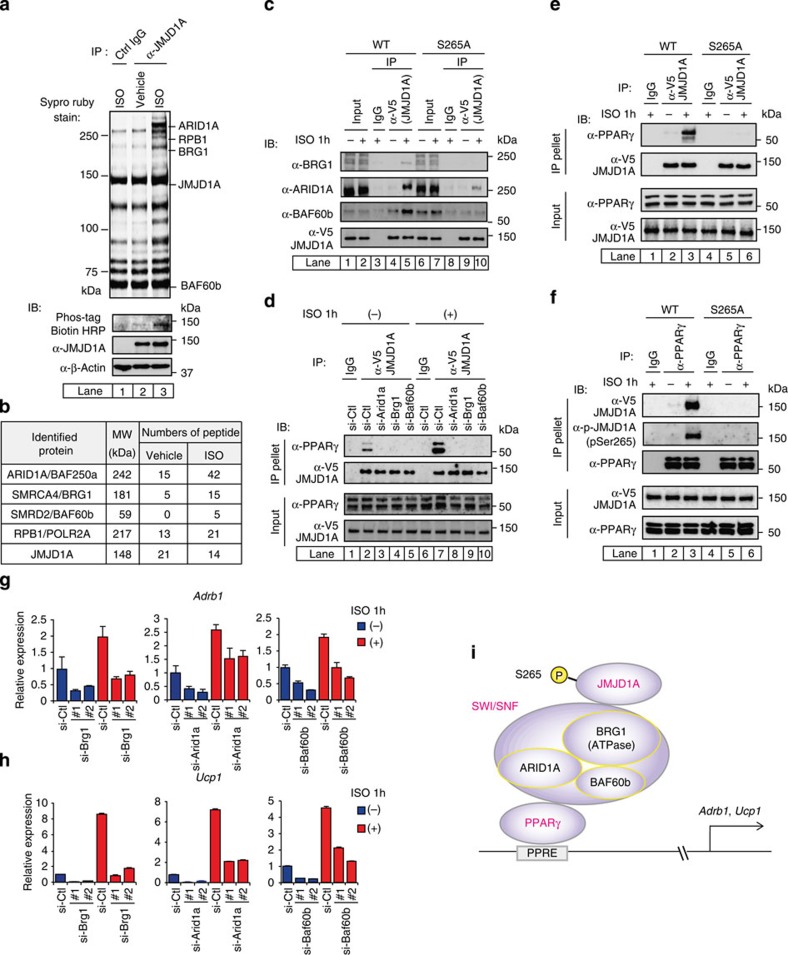
Phosphorylation of JMJD1A triggers the interaction with SWI/SNF and PPARγ. (**a**,**b**) JMJD1A-associated proteins were immunoprecipitated with anti-mJMJD1A antibody (IgG-F0231) from 3T3-L1 cells treated with ISO (10 μM for 1 h), separated by SDS–PAGE gel, stained with SYPRO Ruby and then subjected to in-gel digestion for mass spectrometry (**a**, top panel). Identified proteins were shown in **b** and Supplementary Fig. 4a. P-JMJD1A protein was demonstrated by immunoblot (IB) analysis using anti-phospho-S265-JMJD1A antibody (**a**, bottom panel). (**c**) Nuclear extracts from either WT- or S265A-hJMJD1A-iBAT^sh^s were treated with either ISO (10 μM for 1 h) or vehicle and subjected to immunoprecipitation (IP) with anti-V5 antibody and followed by IB analysis with anti-BRG1, anti-ARID1A or anti-BAF60b. (**d**) ISO-dependent JMJD1A association with PPARγ via ARID1A, BRG1 and BAF60b. WT-hJMJD1A-iBAT^sh^s were subjected to IP with anti-V5 and followed by IB with anti-PPARγ antibody (IgG-A3409). (**e**,**f**) S265 phosphorylation is crucial for JMJD1A binding to PPARγ. WT or S265A-hJMJD1A-iBAT^sh^s were pre-cultured in 0.1% bovine serum albumin containing DMEM for 6 h then treated with ISO (10 μM for 1 h) or vehicle and nuclear extracts from each cells were subjected IP with anti-V5 antibody followed by IB with anti-PPARγ antibody (**e**). The same extracts were also subjected IP with anti-PPARγ antibody followed by IB with either anti-V5 antibody or anti-P-S265-JMJD1A antibody (**f**). (**g**,**h**) JMJD1A and SWI/SNF complex interaction was functionally linked to gene expressions. *Adrb1* and *Ucp1* mRNA levels were quantified by RT-qPCR in WT-hJMJD1A-iBAT^sh^s transfected with control short interfering RNA (siRNA) or two independent siRNAs specifically targeting *Arid1a*, *Brg1* or *Baf60b* under either ISO-plus (1 μM for 1 h) or minus condition. Data were presented as fold change relative to control siRNA transfected cells under ISO-minus condition. Error bars represent mean±s.e.m. of three technical replicates. The experiments were performed at least three times and the most representative one is shown. (**i**) Schematic drawing of JMJD1A–SWI/SNF–PPARγ complex. P-JMJD1A at S265 induces forming a complex with SWI/SNF chromatin remodeler and TF PPARγ recruited to PPRE (Fig. 1b). PPRE, PPAR-responsive element. Uncropped images of the blots (**a**,**c**–**f**) are shown in Supplementary Figs 11 and 12.

To identify co-regulator complexes for P-JMJD1A, nuclear extracts of ISO-treated 3T3-L1 adipocytes were immunoprecipitated with anti-mJMJD1A antibody. Putative interacting partners were separated by SDS–PAGE followed by SYPRO Ruby stain (Fig. 4a). LC/MS/MS analysis led to the identification of three subunits of the SWI/SNF chromatin remodelling complex within the JMJD1A immunoprecipitation complex: AT-rich interactive domain containing protein 1A (ARID1A), BRG1 (also referred to as SMARCA4) and BRG1-associated factor of 60 kDa, subunit b (BAF60b) (Fig. 4b and Supplementary Fig. 4a). The numbers of peptides identified in the immunoprecipitates were significantly increased when the cells were pretreated with ISO (Fig. 4b). BRG1, ARID1A and BAF60b were also detected by immunoblot analysis of the immunoprecipitates of V5-tagged WT-hJMJD1A expressing iBAT^sh^s lysates confirming the LC/MS/MS analysis. Interestingly, the interaction of these SWI/SNF components with JMJD1A was increased by ISO treatment (Fig. 4c, compare lanes 4 and 5). By contrast, SWI/SNF components were recovered at very low levels from the co-immunoprecipitates of S265A-hJMJD1A-iBAT^sh^s lysates (Fig. 4c, compare lanes 9 and 10). The total cellular levels of the SWI/SNF factors were not clearly affected by ISO (Fig. 4c, compare lanes 1 and 2). An interaction between PPARγ and JMJD1A, which was inducible upon ISO treatment of iBATs, was detected as well (Fig. 4d, compare lanes 2 and 7). Following transfection of short interfering RNAs specifically targeting *Arid1a, Brg1, or Baf60b*, co-immunoprecipitation (co-IP) of JMJD1A with PPARγ was no longer observed (Fig. 4d), demonstrating that the association between JMJD1A and PPARγ requires SWI/SNF. We next clarified whether or not only phosphorylated JMJD1A (P-JMJD1A) is able to associate with SWI/SNF complex and PPARγ. Because fetal bovine serum used for cell culture already contains non-negligible levels of catecholamines, we pre-cultured differentiated iBATs for 6 h in 0.1% bovine serum albumin, then added ISO for 1 h and performed a co-IP assay. The result in Fig. 4e showed that PPARγ was co-immunoprecipitated with JMJD1A only under the ISO-plus condition (Fig. 4e, compare lanes 2 and 3). Non-phosphorylated form S265A JMJD1A was not co-immunoprecipitated, indicating that this interaction was dependent on phosphorylation of S265 (Fig. 4e, lanes 5 and 6). JMJD1A was also co-immunoprecipitated with an antibody to PPARγ but only when ISO was included. The JMJD1A bound to PPARγ was indeed phosphorylated because it was detected by the anti-P-JMJD1A antibody (Fig. 4f, compare lanes 2 and 3). These data demonstrate that only P-JMJD1A is able to associates with SWI/SNF complex and PPARγ.

### Crosstalk between SWI/SNF and P-JMJD1A in gene regulation

We next examined the role of SWI/SNF interaction with P-JMJD1A in activation of gene expression for *Adrb1* and *Ucp1*. The basal and ISO-induced levels of *Adrb1* and *Ucp1* were significantly reduced following knockdown of *Arid1a*, *Brg1* or *Baf60b* (Fig. 4g,h). Specificity of the knockdown was validated by qPCR and immunoblot analyses (Supplementary Fig. 4b,c). These data support the model in Fig. 4I, predicting that SWI/SNF is involved in the phosphorylation-dependent activation of gene expression by JMJD1A.

### Co-localization of JMJD1A and SWI/SNF on PPARγ targets

**Figure 5:**
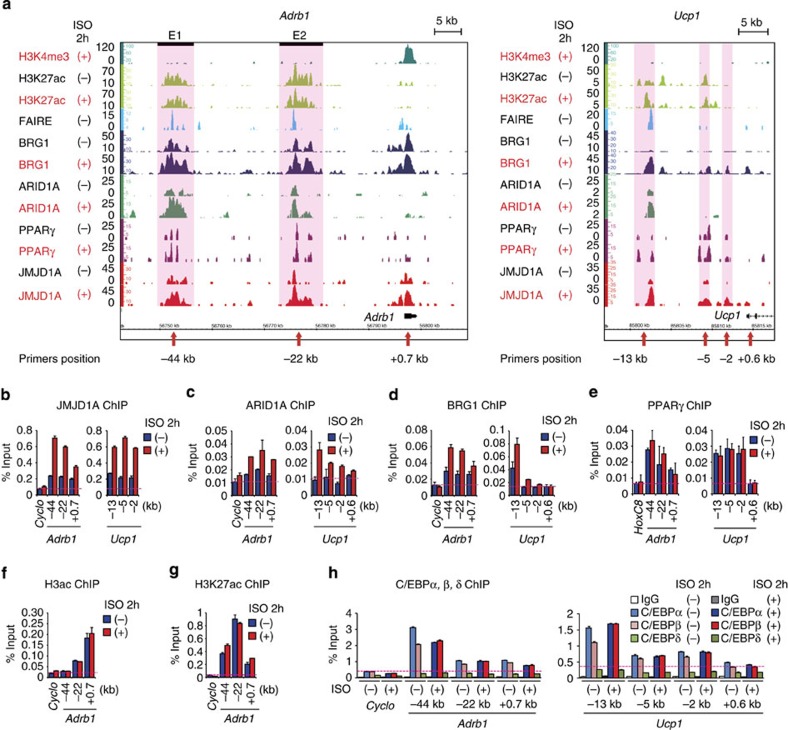
Co-localization of JMJD1A–SWI/SNF-PPARγ across *Adrb1* and *Ucp1* genomic regions. (**a**) ChIP-seq profiles for H3K4me3, H3K27ac, BRG1, ARID1A, PPARγ and JMJD1A and formaldehyde-assisted isolation of regulatory element (FAIRE)-seq open chromatin profile on *Adrb1* and *Ucp1* genomic regions. iBATs (day 8) were treated with 1 μM ISO or vehicle for 2 h and subjected to ChIP-seq or FAIRE-seq analysis. Light pink shadows highlight the enhancers from H3K27ac ChIP-seq data. JMJD1A, SWI/SNF components (ARID1A and BRG1) and PPARγ co-localized at distal enhances of *Adrb1* and *Ucp1.* Scale bars, 5 kb. (**b**–**h**) ChIP–qPCR of ISO-induced binding of JMJD1A (**b**), ARID1A (**c**), BRG1 (**d**), PPARγ (**e**), H3ac (**f**), H3K27ac (**g**), C/EPBα (**h**), C/EBPβ (**h**) or C/EBPδ (**h**) on enhancers each of *Adrb1* and *Ucp1*. Vertical axis represents % input. The experiments in **b**–**h** were performed at least three times and the representative one is shown. Error bars represent mean±s.e.m. of three technical replicates.

Next, we used ChIP-seq for two major components of the SWI/SNF complex (BRG1 and ARID1A; Fig. 5a) and compared the results with our JMJD1A ChIP-seq (Fig. 1c,e and Supplementary Fig. 1b). We also included formaldehyde-assisted isolation of regulatory element-seq (FAIRE-seq) and ChIP-seq for histone H3 Lys-4 tri-methylation (H3K4me3) and histone H3 Lys-27 acetylation (H3K27ac) in control and ISO-treated iBATs (Fig. 5a). The co-localization of JMJD1A, BRG1, ARID1A and PPARγ was revealed by the significant overlap in sequence tag-density profiles comparing the corresponding genome browser tracks shown in Fig. 5a with our ChIP-seq data sets for PPARγ and JMJD1A (Fig. 1c,e and Supplementary Fig. 1b). We also directly compared the binding signals of ARID1A, BRG1, PPARγ and JMJD1A obtained from ChIP-seq data within the top 10,000 binding sites of JMJD1A in iBATs. The heatmap in Supplementary Fig. 5a shows co-localization of ARID1A, BRG1 and PPARγ with JMJD1A on the 39 ISO-stimulated JMJD1A target genes selected in Fig. 1d. Several representative ChIP-seq profiles of their targets and ISO-induced gene expression profiles are also shown in Supplementary Fig. 6. These results strongly suggest that formation of this higher-order complex is a key step for β-adrenergic-induced JMJD1A recruitment to its target genes, which is also a prerequisite for subsequent H3K9me2 demethylation of chromatin at the *Ucp1* enhancer (∼2 kb) as previously shown^14^ (Fig. 4i).

### JMJD1A occupies lineage-specific distal enhancers

Recent studies have shown that SWI/SNF-mediated higher-order chromatin interactions play significant roles in gene transcription^16^. In our studies, ChIP-seq analysis showed that ∼24% of JMJD1A occupied sites are intergenic regions (Fig. 1a). Comparing the JMJD1A localization to H3K27ac, an epigenetic mark of active enhancers revealed that JMJD1A occupied H3K27ac-enriched remote enhancers following ISO treatment. In addition, the ChIP-seq comparison in Fig. 5a showed that PPARγ, BRG1 and ARID1A were also localized to these chromatin regions. These observations prompted us to propose that a JMJD1A–SWI/SNF-PPARγ complex plays a significant role in controlling higher-order chromatin interactions to regulate gene transcription in response to β-adrenergic stimulation. The potential remote enhancers marked by H3K27ac located 20–50 kb upstream of *Adrb1* (Fig. 5a), which is an ideal distance to examine a long-range looping model by chromosome conformation capture (3C) analysis^19^.

A survey of the extended *Adrb1* locus revealed JMJD1A occupancy in the vicinity of *Adrb1* gene body and also to chromatin domains located 44 and 22 kb upstream (Fig. 5a,b, left panel). We refer to 44 and 22 kb elements here as E1 and E2 enhancers, respectively, because they both displayed H3K27ac enrichment and only low levels of H3K4me3 (Fig. 5a), suggesting that they contain active enhancers. An evaluation of published ChIP-seq data obtained from normal mouse tissues also revealed that H3K27ac was present at E1 and E2 regions specifically in brown adipose tissue (Supplementary Fig. 5b, left panel). Further quantification by ChIP–qPCR showed that recruitment of JMJD1A, ARID1A and BRG1 to these two enhancers was increased by ISO treatment (Fig. 5b–d, left panels). By contrast, PPARγ was recruited to these enhancers of *Adrb1* gene regardless of ISO stimulation, indicating that PPARγ is bound before stimulation (Fig. 5e, left panel). ISO treatment did not grossly perturb levels of H3K27ac at E1 and E2 (Fig. 5a, left panel; Fig. 5f,g). Since CCAAT/enhancer binding protein-α (C/EBPα) and C/EBPβ associate with SWI/SNF^20^, we postulated that C/EBP family members may associate with E1 and E2. ChIP–qPCR showed that C/EBPα and C/EBPβ also occupy the E1 and E2 enhancers (Fig. 5h, left panel).

Additional analysis of ChIP-seq and ChIP–qPCR on the *Ucp1* locus showed co-localization of ARID1A, BRG1, PPARγ, C/EBPα and C/EBPβ with JMJD1A, as well as enrichment of H3K27ac at the enhancer region located 13, 5 and 2 kb upstream of the translation initiation site (Fig. 5a–e,h, right panels). An evaluation of published ChIP-seq data obtained from normal mouse tissues also revealed that H3K27ac was present at -13, -5 and -2 kb regions (Supplementary Fig. 5b, right panel).

### P-JMJD1A mediates *Adrb1* enhancer–promoter interaction

**Figure 6:**
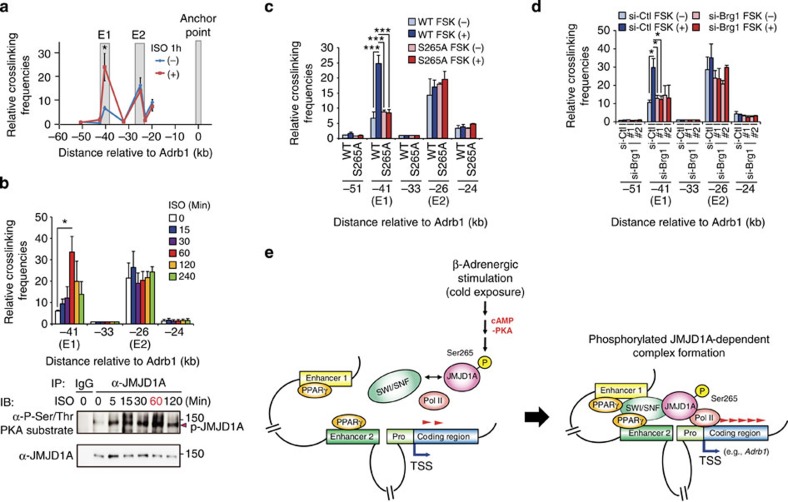
P-JMJD1A mediates PKA-induced enhancer–promoter interaction at the *Adrb1* locus. (**a**–**d**) 3C-qPCR analysis of the interaction frequency of the restriction fragments with the anchor point fixed near the *Adrb1* gene. The grey shadows in **a** highlight the regions containing E1 and E2 enhancer elements and anchor point. Crosslinked chromatin samples were prepared from differentiated iBATs (day 8) treated with 1 μM ISO or vehicle for 1 h (**a**), treated with 1 μM ISO for the indicated time periods (**b**), from WT- and S265A-hJMJD1A iBAT^sh^s treated with 20 μM FSK or vehicle for 20 min (**c**) or from differentiated iBATs transfected with control or two independent *Brg1* siRNA treated with 20 μM FSK or vehicle for 20 min (**d**). Time course of ISO-induced JMJD1A phosphorylation was determined by immunoprecipitation (IP) followed by immunoblot (IB) analysis (**b**, bottom panel). Uncropped images of the blots are shown in Supplementary Fig. 13. Error bars represent±s.e.m. of three independent experiments. Student's *t*-test was performed for comparisons in **a** and analysis of variance were performed followed by Tukey's *post hoc* comparison in **b**–**d**. *P<0.05 and ***P<0.001 were considered statistically significant. (**e**) A schematic model. See the discussion for details.

The above observations raised the intriguing hypothesis that ISO facilitates long-range DNA looping such that TFs (PPARγ, CEBPα and β) and the SWI/SNF chromatin remodelling complex that are located at distal E1 and/or E2 enhancers are brought in proximity to the TSS of *Adrb1*, facilitating the recruitment of RNA polymerase and a subsequent increase in *Adrb1* gene transcription. To evaluate this, we performed a 3C analysis quantified by qPCR using an anchor point fixed near *Adrb1* or E1. 3C-qPCR using the *Adrb1* anchor point revealed that E1 made contact with *Adrb1* and its contact frequency was inducible by ISO treatment (Fig. 6a). 3C-qPCR using an anchor point at E1 showed that both E1-E2 proximity and E1-*Adrb1* promoter proximity were ISO dependent (Supplementary Fig. 7a). Together, these data indicate that E1 is brought in close proximity to the E2-*Adrb1* promoter contact via DNA long-range looping in an ISO-inducible manner as schematically illustrated in Fig. 6e. The looping interactions between E1 and *Adrb1* promoter were detected as early as 15 min and reached a maximum at 60 min and reduced by 60% after 120 min following ISO treatment and then declined (Fig. 6b, top panel). This time course was closely matched by the timing of PKA-dependent phosphorylation of JMJD1A that was detected as early as 5–15 min, peaked at 60 min and was reduced to 60% after 120 min (Fig. 6b, bottom panel). This was also correlated with the pattern of *Adrb1* mRNA accumulation (Fig. 1e). Similar results were obtained following forskolin (FSK) activation of cAMP signalling (Supplementary Fig. 7b).

Interestingly, the S265A mutation significantly reduced the long-range looping signal in response to FSK treatment (Fig. 6c). Similarly, knockdown of *Brg1* abrogated the FSK-dependent chromatin looping between E1 and *Adrb1* promoter as well (Fig. 6d). Collectively, we conclude that PKA-dependent JMJD1A phosphorylation at S265 facilitates higher-order chromatin conformation changes that require SWI/SNF.

### Enhancer–promoter interactions increase *Adrb1* expression

**Figure 7:**
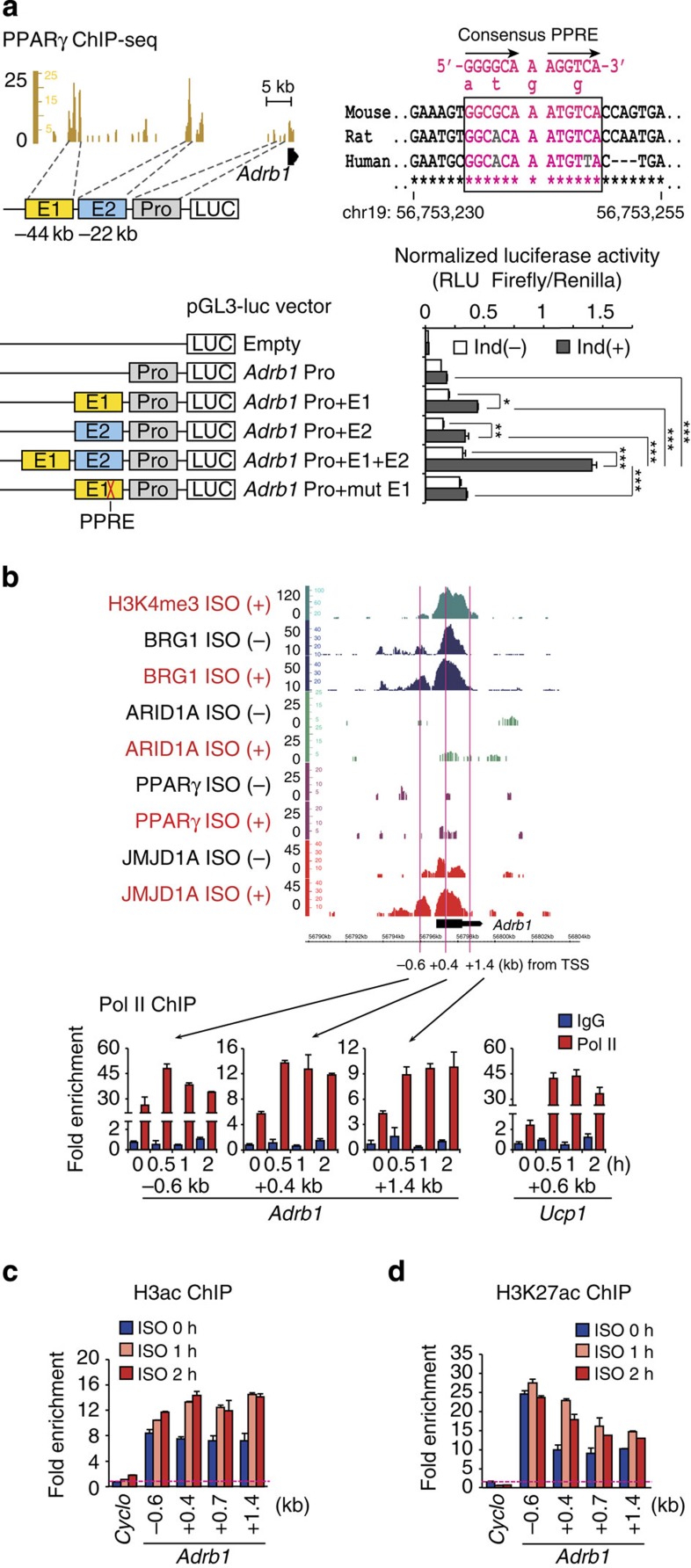
Looping of two enhancers to the *Adrb1* promoter synergistically enhances *Adrb1* gene expression. (**a**) Luciferase reporter activity driven by *Adrb1* promoter and two enhancer elements. iBATs were transfected with the indicated luciferase reporter plasmids (left panel) and cultured with differentiation medium containing 5 μg ml^−1^ insulin plus 125 μM indomethacin or only insulin for 2 days, then the luciferase activity was measured (right bottom panel). Mutated PPRE consensus sequence in Pro+mut E1 plasmid is shown (top right panel). Data were normalized to Renilla internal control. Error bars represent±s.e.m. of three independent experiments. Analysis of variance were performed followed by Tukey's test, and *P<0.05, **P<0.01 and ***P<0.001 were considered statistically significant. (**b**–**d**) ISO-induced RNA Pol II recruitment and histone acetylation mark at the vicinity of *Adrb1* and *Ucp1* genes. ChIP–qPCR analysis of Pol II (**b**), H3ac (**c**) and H3K27ac (**d**) in iBATs at day 8 of differentiation treated with 1 μM ISO for 0, 1 or 2 h at *Adrb1* gene (**b**, top panel). Genome browser shot of the *Adrb1* gene from Fig. 5a and the positions of the sets of primers used for the Pol II ChIP–qPCR are denoted (**b**, top panel). Data are normalized to precipitated DNA (fold enrichment). Error bars represent±s.e.m. of three technical replicates. PPRE, PPAR-responsive element; RLU, relative light unit.

To examine the combined roles for E1 and E2, and looping in *Adrb1* gene expression, the promoter region was cloned with one or both of the two enhancer regions into pGL3 basic luciferase vector and transfected into iBATs (Fig. 7a, left panel) that were then cultured in the absence or presence of a differentiation cocktail. The experiments demonstrated that the promoter had weak activity, and that each of the two enhancers only contributed a modest increase in luciferase reporter expression (∼2-fold) (Fig. 7a). Intriguingly, the combination of both E1 and E2 enhancers synergistically drove luciferase gene expression (eightfold; Fig. 7a). ChIP–qPCR by RNA polymerase II (Pol II) showed that Pol II recruitment at TSS and across *Adrb1* gene body was induced by twofold at 1 h ISO treatment (Fig. 7b, bottom panel). This was accompanied by hyper-acetylation of histone H3 at *Adrb1* gene body (Fig. 7c,d). The induction of Pol II recruitment was also observed on the *Ucp1* gene body (20-fold in Fig. 7b). Agreement with previous study, these data indicate that mRNA levels are due to increased transcriptions rather than mRNA stability under β-adrenergic stimulation^21^.

Luciferase reporter analysis on the *Ucp1* locus showed that pGL3 luciferase reporter construct that harbours the proximal (−2 kb) enhancer–promoter drove appreciable level of luciferase gene expression and the additional two distal enhancers (−13 and −5 kb regions) plus proximal (−2 kb) enhancer–promoter to this construct drove much higher luciferase gene expression (Supplementary Fig. 8). This result suggests that distal enhancers are important in *Ucp1* gene expression. Since the S265A mutation in JMJD1A severely reduced *Ucp1* expression (Fig. 3a), it suggests that P-JMJD1A–SWI/SNF-PPARγ may also contribute to long-range DNA looping at *Ucp1* to facilitate enhancer–promoter interactions.

### P-S265-JMJD1A regulates thermogenesis

Among P-JMJD1A targets, ADRB1 is the most upstream component in the β-adrenergic signalling pathway. As reported previously^21^, the ISO-induced *Adrb1* gene expression reached a peak at 1 h (Fig. 1e). This was also reflected at ADRB1 protein levels (Supplementary Fig. 9a). We also showed that expression of *Ucp1*, another P-JMJD1A target, was also reduced at the mRNA and protein levels in S265A-hJMJD1A-iBAT^sh^s (Fig. 3a and Supplementary Fig. 9a). Maximum cAMP production following treatment with the ADRB1 selective agonist dobutamine (DOB) in S265A-hJMJD1A-iBAT^sh^s was reduced relative to identically treated WT-hJMJD1A-iBAT^sh^s (Supplementary Fig. 9b), which is consistent with reduced ADRB1 protein in the S265A-hJMJD1A-iBAT^sh^s (Supplementary Fig. 9a). Also, DOB-stimulated glycerol release was blunted in S265A-hJMJD1A-iBAT^sh^s (Supplementary Fig. 9c). To examine further whether phosphorylation of S265 is critical for BATs function, we measured respiratory activity using the extracellular flux analyser. The mitochondrial respiration of WT-hJMJD1A-iBAT^sh^s was markedly induced (approximately double) by DOB stimulation, while this induction was markedly blunted in S265A-hJMJD1A-iBAT^sh^s (Supplementary Fig. 9d). This DOB-induced respiration in WT-hJMJD1A-iBAT^sh^s was largely insensitive to oligomycin and was thus uncoupled (Supplementary Fig. 9d, top panels). The characteristic proton leakage profile in BATs was indeed reduced in S265A-hJMJD1A-iBAT^sh^s (Supplementary Fig. 9d). These additional observations demonstrate that the key cellular responses mediated by β1-AR signalling are also reduced in response to the S265A-JMJD1A mutation.

### P-S265 JMJD1A in brown adipose tissue of mice *in vivo*

**Figure 8:**
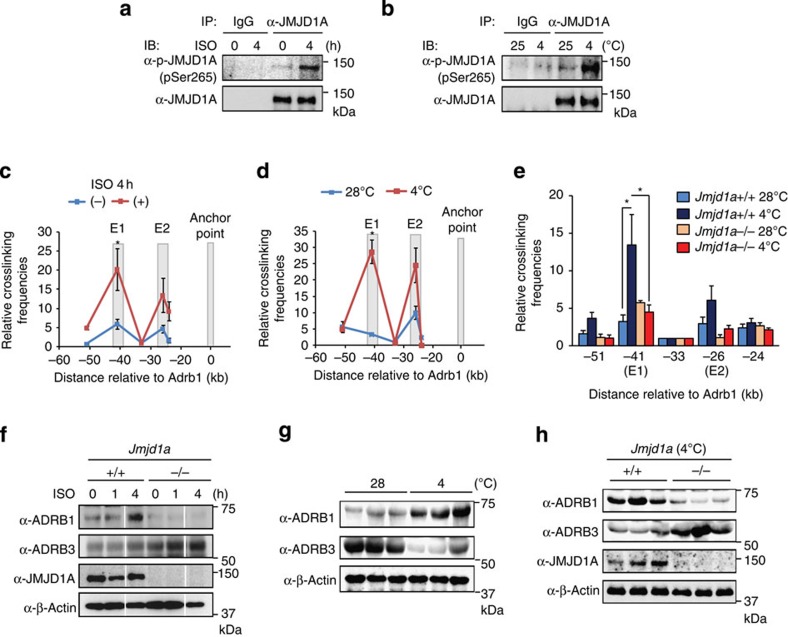
P-S265-JMJD1A induces enhancer–promoter interaction in response to β-adrenergic signalling in brown adipose tissue of mice *in vivo*. (**a**,**b**) Immunoblot (IB) analyses for P-JMJD1A proteins in the brown adipose tissue from 14-week-old C57BL/6J mice treated with ISO (10 mg kg^−1^, by subcutaneous (s.c.) injection) for 4 h (**a**) or 12-week-old C57BL/6J mice placed at 25 or 4 °C for 6 h (**b**). Whole-cell extracts from brown adipose tissue were subjected to immunoprecipitation (IP) followed by IB analysis. (**c**–**e**) Dynamic changes in higher-order chromatin conformation of the *Adrb1* locus in brown adipose tissue of ISO-induced and cold-exposed mice. 3C-qPCR analysis was performed with the anchor point fixed near the *Adrb1* gene in brown adipose tissue of *Jmjd1a+/+* mice injected with ISO (10 mg kg^−1^, by s.c. injection) for 4 h (**c**) or exposed to 28 or 4 °C for 6 h (**d**), or *Jmjd1a+/+* and *Jmjd1a−/−* mice exposed to 28 or 4 °C for 6 h (**e**) as described in Fig. 6a–d. Error bars represent±s.e.m. of three independent experiments. Student's *t*-test was performed for comparisons in **c** and **d**, and analysis of variance were performed followed by Tukey's *post hoc* comparison in **e**. *P<0.05 was considered statistically significant. (**f**–**h**) IB analysis for ADRB1 and ADRB3 proteins in brown adipose tissue from *Jmjd1a+/+* or *Jmjd1a−/−* mice injected with ISO (10 mg kg^−1^, by s.c. injection) for indicated hours (**f**), from WT mice placed at 28 or 4 °C for 6 h (*n*=3 mice per group; **g**), and brown adipose tissue from *Jmjd1a+/+* and *Jmjd1a−/−* mice exposed to 4 °C for 6 h (*n*=3 mice per group; **h**). Uncropped images of the blots (**a**,**b**,**f**–**h**) are shown in Supplementary Fig. 13.

To analyse the physiological relevance of the above observations, we evaluated the phosphorylation switch model for JMJD1A *in vivo*. First, using the anti-P-S265-JMJD1A antibody, we showed that JMJD1A was phosphorylated in brown adipose tissue of mice *in vivo* in response to both ISO injection and following cold exposure (Fig. 8a,b). *Jmjd1a−/−* mice are cold intolerant (Supplementary Fig. 10a and ref. 14) and exhibit lower oxygen consumption relative to *Jmjd1a+/+* mice under cold exposure (4 °C) (Supplementary Fig. 10b). We next used the 3C method and showed that the enhancer–promoter proximity between E1 enhancer and *Adrb1* promoter was significantly increased in brown adipose tissue following ISO treatment and cold exposure in *Jmjd1a+/+* mice but not in identically treated *Jmjd1a−/−* mice (Fig. 8c–e). We also measured *Adrb1* protein levels and showed that ISO and cold exposure selectively increased ADRB1 expression that was selectively increased in the *Jmjd1a+/+* mice, whereas ADRB3 was constitutively elevated in *Jmjd1a−/−* mice but was unaffected by ISO (Fig. 8f–h). This reciprocal effect is consistent with previous reports^21^. These results indicate that JMJD1A is required for enhancer E1-*Adrb1* promoter proximity in β-adrenergic stimulation and validated the observations in iBATs culture system.

Taken together, these results provide strong evidence that S265 of JMJD1A is indeed phosphorylated *in vivo* by β-adrenergic stimulation and participates in enhancer–promoter looping in a physiologically relevant context.

## Discussion

In the current study, we have identified JMJD1A as a signal-sensing scaffold that transduces thermogenic cAMP signalling to activation of target gene expression through rapid changes in higher-order chromatin organization. The molecular basis of signal sensing by JMJD1A is attributable to direct PKA-dependent phosphorylation at S265. Using an antibody that specifically recognizes the S265 phosphorylation, we showed that JMJD1A was phosphorylated at this site in response to β-adrenergic stimulation in cultured iBATs and in brown adipose tissue *in vivo*. Mechanistically, we showed that assembly of the JMJD1A–SWI/SNF complex, recruitment of signature enhancers to a target promoter and transcriptional activation were all dependent on PKA phosphorylation of S265 in JMJD1A. As a result of this PKA-dependent switch in JMJD1A–SWI/SNF interaction, a PPARγ-bound enhancer is brought in proximity of the responsive TSS, which accelerates RNA polymerase recruitment and transcription (Fig. 6e). Thus, a JMJD1A–SWI/SNF–PPARγ complex serves as a cAMP-sensing epigenetic determinant that couples DNA long-range looping and transcription of thermogenic genes. Whereas PKA-dependent phosphorylation was required for JMJD1A to interact with SWI/SNF, its demethylase activity was not affected by phosphorylation and a mutation that destroyed demethylase activity did not affect JMJD1A functional interaction with SWI/SNF and PPARγ. Therefore, the role of JMJD1A as a regulator of enhancer–promoter interactions at *Adrb1* is separate from its role in H3K9 demethylation.

The *Adrb1* gene product (β1-AR) is the most upstream component of β-adrenergic signalling pathway that directly couples the cAMP-PKA-mediated phosphorylation cascade with downstream cellular events. We identified *Adrb1* as a *bona fide* target of cAMP-sensing through JMJD1A. *Adrb1* is under positive β-adrenergic control and is thus self-inducing under the condition of increased sympathetic tone^21^,^22^. Importantly, the response to norepinephrine through increased *Adrb1* is inherently transient because its transcript quickly decays with an mRNA half-life of 17 min (ref. 21). This rapid ‘On-Off' regulation by extracellular cues must be flexible and adaptive to be physiologically relevant to responses such as thermogenesis, which requires immediate and robust fuel mobilization following acute cold exposure. It is equally important for this positive feedback control to be promptly cancelled once the cAMP levels reaches higher unfavourable levels in the target cells^23^.

One important characteristic of the machinery revealed in the current study is the PKA-dependent ‘spatial regulation' of the remote enhancer and associated TFs, which conferred the rapid ‘On-Off' transcriptional switch. Once the extracellular signal is sensed, transcriptionally competent TFs (for example, PPARγ, CEBPα and CEBPβ) waiting at remote enhancers are immediately recruited to the promoter/TSS via long-range looping to turn transcription ‘On'. On the flip side, enhanced transcription can also be quickly turned ‘Off' via the rapid dephosphorylation of JMJD1A and the disassembly of the JMJD1A–SWI/SNF–PPARγ complex. This spatial regulation of remote enhancer and associated competent proteins (TFs and chromatin remodelers) may have profound merit for a rapid on-off gene transcriptional switch.

It has been postulated that β3-AR plays a leading role in activation of brown adipose tissue^24^. However, Bengtsson *et al*.^21^,^25^ reported that acute cold exposure of mice led to full cessation of expression of *Adrb3* but a reciprocal significant increase in *Adrb1*. Our results show a similar reciprocal expression pattern for the two different β-AR proteins in response to cold in WT mice (Fig. 8g). Mice with a global β3-AR knockout (β3KO) have no significant defect in adaptive response to cold exposure^26^,^27^. These animals do exhibit upregulation of β1-AR (not β2), suggesting that its absence is compensated by β1-AR. In addition, β1-AR KO (β1KO) alone reduced brown adipose tissue mass and thermogenic responsiveness^28^. Taken together, all of the available data indicate that β1-AR is important for thermogenesis and the new results of the current study show that JMJD1A is pivotal for β-adrenergic signalling, activation of *Adrb1* expression and thermogenesis.

*Ucp1* gene expression was severely reduced in iBATs that express the S265A mutant JMJD1A. Upstream of *Ucp1* gene, we found three distinct enhancers that exhibited an increase in H3K27ac and binding by PPARγ after ISO treatment. Recruitment of both JMJD1A and also SWI/SNF proteins were also induced by ISO.

The PKA-dependent scaffold function of JMJD1A is independent of its lysine demethylase function. Previous study showed that JMJD1A is recruited to the proximal enhancer (∼2 kb) of *Ucp1* gene and decreases levels of H3K9me2 in cultured BATs^14^. However, the mechanism for the β-adrenergic-dependent recruitment of JMJD1A to the *Ucp1* enhancers was elusive. In the current study, we show that association of P-JMJD1A with SWI/SNF and PPARγ conferred β-adrenergic-induced JMJD1A recruitment to target sites. Thus, the S265 phosphorylation on JMJD1A is possibly a prerequisite to recruit the enzyme to genomic target sites where the local chromatin serves as a substrate for demethylation of H3K9me2. Chronic conditions such as prolonged cold exposure (for example, several days to weeks exposure) or cold exposure after acclimating at thermoneutral conditions may lead to more drastic H3K9me2 changes at enhancers in brown fat as a consequence of the chronic adaption to cold environment.

Overall, the current studies combined with previous observations suggest that JMJD1A has dual roles for the adaptation to environmental cues (for example, catecholamine): one for hormone-induced dynamic chromatin remodelling and the other for demethylation that ensures a long-term stable pattern of gene expression. Our data suggest that PKA-dependent DNA looping plays a more dominant role for the acute and dynamic response, while H3K9 demethylation subsequently plays a key role for a more stable long-term change in gene expression.

## Methods

### Antibodies

Mouse monoclonal antibodies immunoglobulin G F0618 (IgG-F0618) and IgG-F0231 against mouse JMJD1A (a.a. 843–893) were produced by immunizing separate mice with gp64 fusion protein expressing baculovirus. Mouse monoclonal antibodies IgG-F0026 and IgG-F1628 against hJMJD1A were produced by immunizing mice with affinity-purified recombinant hJMJD1A protein. A rabbit polyclonal phospho-specific antibody against JMJD1A-Ser265 was produced by Operon Biotechnologies with a synthetic phosphopeptide corresponding to residues surrounding Ser265 JMJD1A (Ac- C-KRKS(pS)ENNGS-amide). A list of the other antibodies used in this article is shown in the Supplementary Table 1.

### Immunoprecipitation and immunoblotting

Whole-cell extracts were separated into soluble supernatant, nuclear extracts and the cytosolic fractions from the cells that were prepared as previously described^29^ with slight modifications: we used both protease inhibitors (5 μg ml^−1^ pepstatin A, 10 μg ml^−1^ leupeptin, 2.8 μg ml^−1^ aprotinin, 1 mM dithiothreitol (DTT) and 0.5 mM phenylmethylsulfonyl fluoride (PMSF)) and phosphatase inhibitors (40 mM NaF and 1 mM Na_3_VO_4_). For immunoprecipitation, whole-cell lysates or nuclear extracts were immunoprecipitated in cell lysis buffer (50 mM HEPES-KOH, pH 7.9, 150 mM NaCl, 1.5 mM MgCl_2_ and 1% NP-40) containing both protease inhibitors (5 μg ml^−1^ pepstatin A, 10 μg ml^−1^ leupeptin, 2.8 μg ml^−1^ aprotinin, 1 mM DTT and 0.5 mM PMSF) and phosphatase inhibitors (40 mM NaF and 1 mM Na_3_VO_4_) for 6 h wheel rotating at 4 °C as described, using the antibodies described in the legend to figures. For immunoblot analysis, aliquots of proteins were separated by SDS–PAGE and transferred to nitrocellulose membrane (Bio-Rad Laboratories). Immunodetection was carried out with the indicated antibodies (Supplementary Table 1) and bound antibodies were visualized with peroxidase-conjugated affinity-purified donkey anti-mouse or anti-rabbit IgG using ECL Plus (Amersham Biosciences), and luminescence images were analysed by ImageQuant LAS mini (GE Healthcare). Uncropped images of blots are shown in Supplementary Figs 11–15.

### Chromatin immunoprecipitation

For ChIP using JMJD1A, ARID1A, BRG1, C/EBPα, C/EBPβ and C/EBPδ antibodies, iBATs were crosslinked with 1.5 mM ethylene glycol bis(succinimidylsuccinate) (Thermo Scientific) for 30 min at room temperature (RT) and then directly a second crosslinking was performed by addition of 1% formaldehyde for 10 min. Fixation was stopped by adding 0.2 M glycine. After crosslinking, nuclear pellets were prepared for ChIP as described previously with the following modifications as described before^8^,^29^,^30^. Nuclear pellets were re-suspended in 2 ml lysis buffer (25 mM Tris-HCl, pH 8.0, 3 mM EDTA, 0.2% SDS, 0.9% Triton X-100 and 133 mM NaCl) and chromatin DNA was sheared to 2 kb average in size through sonication. Pre-washed magnetic Dynabeads (Life Technologies) were incubated with a specific antibody listed in Supplementary Table 1 in 500 μl of buffer A (PBS containing 0.01% Tween 20) for 1 h by wheel rotating at RT. Subsequently, sonicated crosslinked nuclear lysates (350 μg in 200 μl buffer A) were added and incubated overnight at 4 °C by wheel rotating. The beads were washed several times and eluted with elution buffer (1% SDS and 100 mM NaHCO_3_), the eluent was incubated with pronase (1 mg ml^−1^) at 42 °C for 2 h and then incubated at 65 °C overnight to reverse the crosslinks. DNA was purified using QIA quick PCR purification kit (Qiagen) and the concentration was measured by Qubit double-stranded DNA High Sensitivity assay kit (Invitrogen). For ChIP using the other antibodies (anti-H3K4me3, anti-H3K9me2, anti-H3K27ac, anti-H3ac, anti-Pol II and anti-PPARγ), iBATs at day 8 of differentiation were crosslinked with 0.5% formaldehyde for 10 min and chromatin DNA was sonicated to be 0.5 kb average in size. ChIP-sequencing was done with an Illumina/Solexa sequencer as previously described^8^,^29^,^30^.

### ChIP–qPCR analysis

ChIP samples were also analysed by gene-specific qPCR analyses. The results were presented as input per cent or normalized to precipitated DNA (fold enrichment). All primer sequences used in this article are listed in Supplementary Table 2.

### Real-time qPCR

The method for qPCR has been described^31^,^32^. PCR was carried out in 384-well plates using the ABI PRISM 7900HT sequence detection system (Applied Biosystems). All reactions were done in triplicate. All primer sequences used in this article are listed in Supplementary Tables 2–4.

### Formaldehyde-assisted isolation of regulatory elements

Formaldehyde-assisted isolation of regulatory elements was performed as described previously^33^,^34^ Briefly, formalin-crosslinked cell pellets were lysed in SDS lysis buffer (50 mM Tris-HCl, pH 8.0, 10 mM ETDA, 1% SDS and proteinase inhibitor cocktail (Nacalai Tesque)) and sonicated by ultrasound homogenizer (Bioruptor UCD-200TM, Cosmo Bio). Samples were subjected to phenol/chloroform extraction twice followed by ethanol precipitation and subsequently purified using QIAquick PCR purification kit (Qiagen).

### ChIP-seq data processing

All bound DNA fragments were mapped to UCSC build mm9 (NCBI Build 37) assembly of the mouse genome by a mapping programme ELAND (http://support.illumina.com/sequencing/sequencing_software/casava.html) based on the 5′-side 36 bp sequences.

### Identifying ChIP-seq-enriched regions

Regions of ChIP-seq that were enriched over the background were identified with SICER^35^,^36^ using the default values for the parameters: Window Size, 200 bp; Gap Size, 400 and E-value threshold, 100. Through this analysis, 27,396 genomic regions were picked up from JMJD1A ChIP-seq in iBATs at day 8 of differentiation under ISO-plus condition and were determined to be ‘significant binding sites' and were used for the following clustering analysis.

### Annotation of the JMJD1A binding sites

The JMJD1A binding sites were annotated as follows: the intergenic binding sites were assigned to the closest genes and the intragenic bindings sites were assigned to those genes.

### Clustering analysis of JMJD1A binding sites

To adjust the distribution of the number of DNA sequence tags mapped in the top 10,000 ‘significant binding sites' of JMJD1A, *Z*-scores of either JMJD1A, ARID1A, BRG1 or PPARγ ChIP-seq were calculated along JMJD1A ChIP-seq in the iBATs under ISO-plus condition. Subsequently, the JMJD1A binding sites were classified by a hierarchical clustering program (HOPACH, http://stat-www.berkeley.edu/~laan/Research/Research_subpages/Papers/hopach.pdf) based on the *Z*-scores.

### Analysis of TF binding motifs

To gain insights into the mechanism by which JMJD1A recognizes is recruited to its cognate DNA sequences, we studied the frequency of known TF binding motifs in the JMJD1A-associated regions with the top 10,000 highest JMJD1A-associated regions with higher SICER scores. DNA sequences that JMJD1A bound to were examined for motifs that are known to be binding sites of TFs using Genomatix (http://www.genomatix.de/). The top 10 known TF binding motifs were selected as the putative binding motifs based on the *Z*-scores representing scores of motif enrichment on the basis of random promoter sequences. To reconstruct exact binding motifs, all sequences corresponding to the identified putative motifs were assembled using a scoring method named matrix similarity score (MSS)^37^.

### Transcriptional microarray analysis

Genome-wide transcriptome analysis was performed using the Affymetrix GeneChip system according to the manufacturer's instructions as we previously described^29^. Labelled cRNA probes were hybridized to Mouse Genome 430 2.0 array (Affymetrix) and scanned by Affymetrix GeneChip scanner 3000 (Affymetrix). To calculate the average difference for each gene probe, GeneChip Analysis Suite software version 5.0 was used (Affymetrix). All transcripts with a detective call of absent across all time points as well as these with average difference call below 100 were excluded from the study. Total RNA was extracted from iBATs at day 8 of differentiation at the indicated time points of ISO treatment (0, 1 and 2 h).

### Database search for identifying kinase consensus sequences

Putative PKA phosphorylation sequences located in JMJD1A were surveyed by the Scansite Motif Scanner (http://scansite.mit.edu/motifscan_seq.phtml).

### In-gel digestion and LC/MS/MS

JMJD1A and/or JMJD1A-associated proteins that were immunoprecipitated from HeLa cells or 3T3-L1 cells treated with ISO (10 μM for 1 h) with anti-hJMJD1A (IgG-F0026) or anti-mJMJD1A (IgG-F0231), respectively, were separated on SDS–PAGE, stained by SYPRO Ruby (Invitrogen), and the excised band was digested in-gel as previously described^38^. LC/MS/MS was performed using an LTQ orbitrap XL ETD mass spectrometer (Thermo Fisher Scientific). The methods for LC/MS/MS were as we described previously^39^, and the subsequent database search for peptide identification was slightly modified from those described previously^39^. Tandem mass (MS/MS) spectra were extracted using Proteome Discoverer version 1.3. All MS/MS samples were analysed using Mascot (Matrix Science, version 2.4.1). Mascot was set up to search Sprot_2013_11.fasta (selected for Mus, 16,693 entries) with the digestion enzyme trypsin, the maximum number of missed cleavage sites of three, fragment ion mass tolerance of 0.60 Da and a precursor ion tolerance of 5.0 p.p.m. Carbamidomethyl of cysteine was specified as a fixed modification and acetylation of the protein N terminus, pyroglutamation of glutamine and phosphorylation of tyrosine, serine and threonine were specified as variable modifications. Scaffold software (version Scaffold 4.2.1, Proteome Software Inc.) was used for peptide/protein identification. Peptide identifications were accepted if they could be established at >95.0% probability, and protein identifications were accepted if they could be established at >99.0% probability and contained at least two identified peptides.

### Establishment of a BAT line and Oil red O staining

Stromal vascular fractions (SVFs) were isolated from interscapular BATs of C57BL/6 mouse at P3 by digesting the brown adipose tissue depots with collagenase D (Roche) and dispase II (Roche) in PBS supplemented with 10 mM CaCl_2_ according to the method described previously^40^. SVFs were subsequently infected with retrovirus expressing large T antigen pBabe SV40 Large T antigen from Addgene (no. 13970) for 12 h. After 3 days of infection, the infected SVFs were selected by puromycin at 2 μg ml^−1^. The immortalized cell line (pre-iBATs) was tested for differentiation capacity and expression levels of *Ucp1* as described^40^. Differentiation into iBATs was induced by the treatment of subconfluent cell first for 2 days insulin (5 μg ml^−1^) and indomethacin (125 μM) in basal medium. The cells were then returned to the basal medium containing insulin (5 μg ml^−1^; maintenance medium), which was replenished every other day. The cells were stained with Oil Red O as described^29^,^30^.

To establish shRNA-mediated JMJD1A knockdown iBATs (iBAT^sh^s), iBATs were transduced with retroviral vector expressing shRNA targeting mouse *Jmjd1a* and were selected by G418 (1 mg ml^−1^) in basal medium for 6 days. To establish iBAT^sh^s ectopically expressing V5-tagged WT or mutants JMJD1A, iBAT^sh^s were infected with retrovirus expressing WT, S264A, S265A, S341A, 3SA, H1120Y, H1120F -hJMJD1A or empty vector followed by Zeocin selection (0.1 mg ml^−1^) for 12 days. The resultant iBATs transformants were designated WT, S264A, S265A, S341A, 3SA, H1120Y, H1120F -hJMJD1A-iBAT^sh^s and empty -hJMJD1A-iBAT^sh^s, respectively.

### Other cell culture

3T3-L1 pre-adipocytes and HeLa cells obtained from American Type Culture Collection and Plat E packaging cells were maintained in basal medium.

### 3C assay

3C assay was performed following a protocol as described previously^41^ with some modification. Briefly, cells were fixed in 1% formaldehyde for 10 min at RT. The crosslinking reaction was quenched with 0.125 M glycine and cells were collected by centrifugation at 400*g* for 5 min at 4 °C. The pellet was suspended in 1 ml of ice-cold lysis buffer (10 mM Tris-HCl, pH 8.0, 10 mM NaCl and 0.2% NP-40) containing protease inhibitors (5 μg ml^−1^ pepstatin A, 10 μg ml^−1^ leupeptin, 2.8 μg ml^−1^ aprotinin, 1 mM DTT and 0.5 mM PMSF) and phosphatase inhibitors (40 mM NaF and 1 mM Na_3_VO_4_), and incubated for 90 min at 4 °C. The swollen cells were centrifuged at 3,300*g* for 1 min at 4 °C in microcentrifuge. The pellet was dissolved in digestion buffer containing 0.3% SDS, incubated for 1 h at 37 °C, digested with 600U Hind III (Takara) containing 1.8% Triton X-100 overnight at 37 °C, followed by random re-ligation with 350U T4 DNA ligase (Takara). After reversing crosslinks by incubation with proteinase K (0.1 mg ml^−1^) at 65 °C for 4 h, genomic DNA was purified by phenol/chloroform extraction and ethanol precipitation. The ligated DNA (50 ng) was assessed by qPCR using ABI 7900HT system (Applied Biosystems) and TaqMan Universal PCR Master Mix (Applied Biosystems). All primer sequences used in this article are listed in Supplementary Table 4. A mouse BAC clones (RP23-469J23) was used to normalize the levels of 3C products that may have been PCR amplified with different primer efficiencies as previously described^42^. For 3C assay using the mouse BATs, fresh tissues were minced in PBS, crosslinked with 1% formaldehyde, homogenized with Dounce-type homogenizer containing 1 ml of ice-cold lysis buffer containing protease inhibitors and phosphatase inhibitors, incubated for 90 min at 4 °C and were centrifuged at 3,300*g* for 1 min at 4 °C. The pellet was processed for Hind III digestion as described above.

### Statistics

All data are presented as mean±s.e.m. Student's *t*-test was performed for the comparison of two groups. For multiple comparisons, analysis of variance were performed followed by Tukey's *post hoc* comparison. *P<0.05, **P<0.01 and ***P<0.001.

## Author contributions

T.I. and J.S. designed the study and wrote the paper. Y.A., R.R., Y.M., T.K., Y.T, K.T.-I., A.S., K.M., K.N. and S.O. carried out experiments. R.N carried out analyses. S.K. and H.K. provided materials.

## Additional information

**Accession codes:** The GEO accession numbers for gene expression microarray data and ChIP-seq data reported in this paper is GSE58936 and GSE67586, respectively.

**How to cite this article:** Abe, Y. *et al*. JMJD1A is a signal-sensing scaffold that regulates acute chromatin dynamics via SWI/SNF association for thermogenesis. *Nat. Commun.* 6:7052 doi: 10.1038/ncomms8052 (2015).

## Supplementary Material

Supplementary Information Supplementary Figures 1-15, Supplementary Tables 1-4, Supplementary Methods and Supplementary References
