# Supplementary material for: JMJD1A is a signal-sensing scaffold that regulates acute chromatin dynamics via SWI/SNF association for thermogenesis
Source: Nat Commun. 2015 May 7;6:7052. doi: 10.1038/ncomms8052 (PMC4432656; doi:10.1038/ncomms8052)
Supplement: Supplementary Information — Supplementary Figures 1-15, Supplementary Tables 1-4, Supplementary Methods and Supplementary References [file ncomms8052-s1.pdf]

Supplementary Information

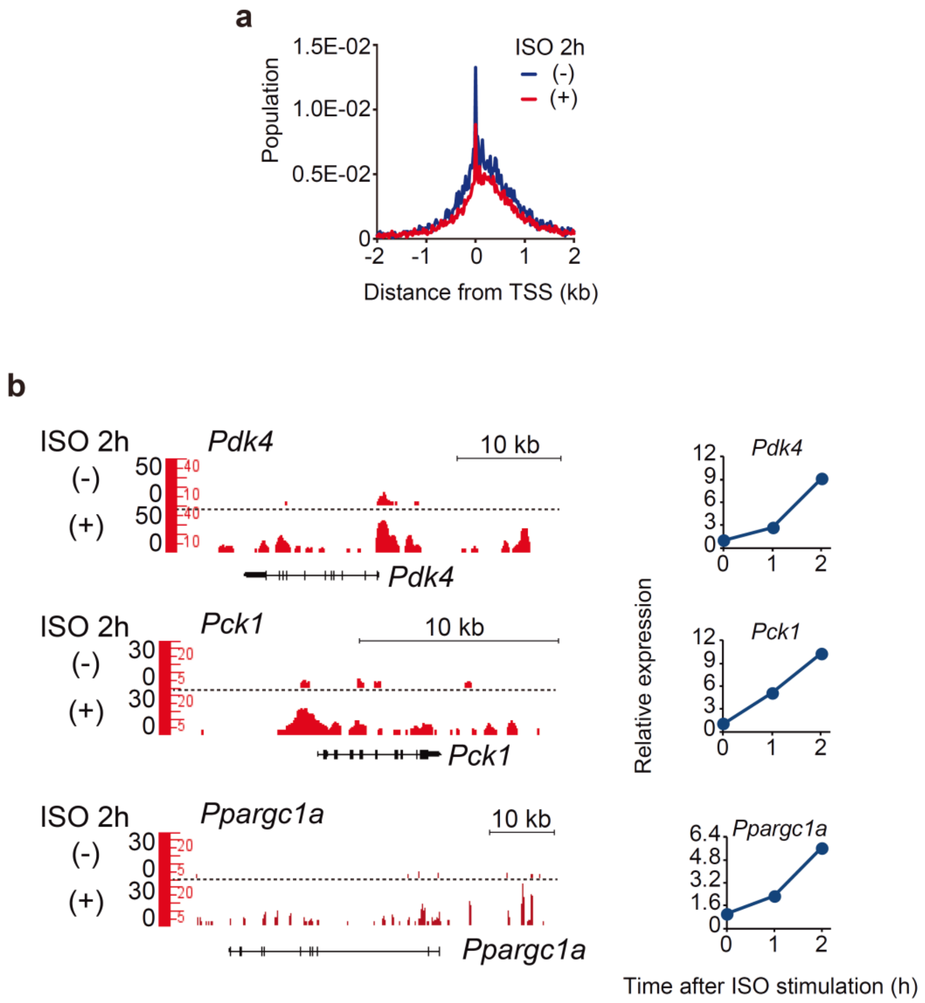

Supplementary Figure 1 | β-Adrenergic dependent genomic localization of JMJD1A. (**a**) ChIP-seq tag distribution of JMJD1A surrounding TSS of JMJD1A targets. TSS, transcription start site; ISO, isoproterenol. (**b**) Genome browser shots showing the ISO induced JMJD1A recruitments on selected genomic regions analyzed by ChIP-seq in iBATs (day 8) treated with ISO or vehicle for 2 hr (left panel). Quantification of mRNA levels by RT-qPCR in iBATs (day 8) at indicated time points after ISO treatment (1 μM). Data were presented as fold change relative to 0 hr (± SEM) of three technical replicates (error bars are too tiny to see) (right panel).

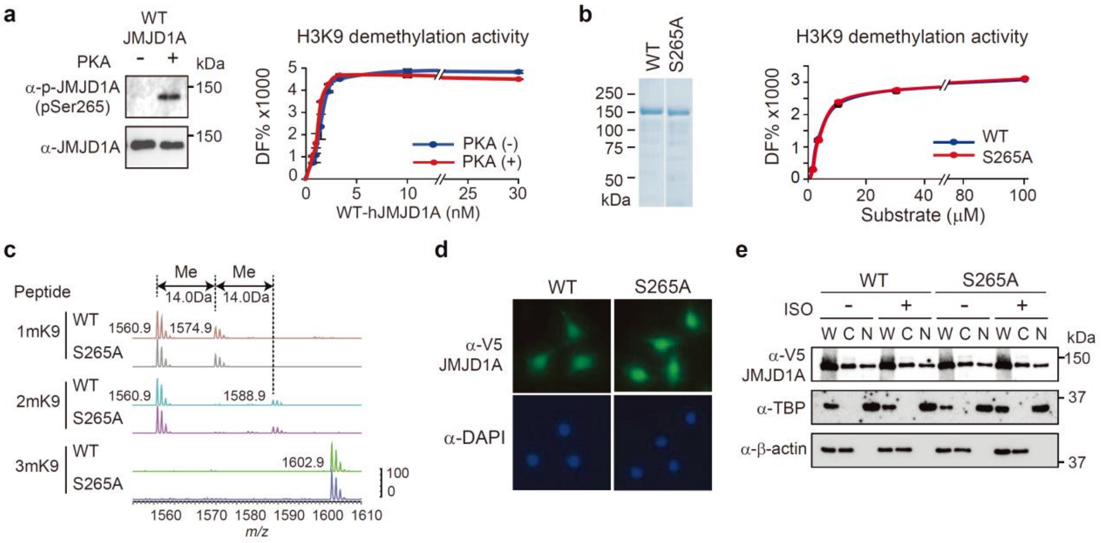

Supplementary Figure 2 | S265-phosphorylation is not essential for H3K9-demethylation activity in vitro. (**a**) Histone demethylase activity of JMJD1A examined by HTRF-FRET histone demethylase assay using either PKA- or vehicle-treated JMJD1A proteins purified from Sf9 cells (right panel). Vertical axis represents DF% as described in the Supplementary Methods. Data are mean ± SD of two technical replicates. P-S265-JMJD1A levels induced by PKA treatment were detected by immunoblot analysis (left panel). (**b**) Histone demethylase activity of WT or S265A JMJD1A determined as described in (**a**) (right panel). The same amounts of both WT and S265A JMJD1A proteins were separated by SDS-PAGE gel (left panel). (**c**) Substrate specificity of WT and S265A JMJD1A determined by MALDI-TOF/MS using H3K9me1, H3K9me2, or H3K9me3. (**d**) Cellular localization of V5-tagged JMJD1A was determined by immunofluorescence analysis in WT- and S265A-hJMJD1A-iBAT^{sh}s using anti-V5 antibody. DAPI was used for DNA counterstaining. (**e**) Subcellular fractionation of WT- and S265A-hJMJD1A iBAT^{sh}s treated with ISO (10 μM) or vehicle for 1 hr demonstrated the localization of JMJD1A in whole cell extracts (W), cytosolic fractions (C) and nuclear extracts (N). β-actin and TBP were used for subcellular markers for cytosolic fractions and nuclear extracts, respectively. Uncropped images of the blots (**a**,**e**) are shown in Supplementary Fig. 14.

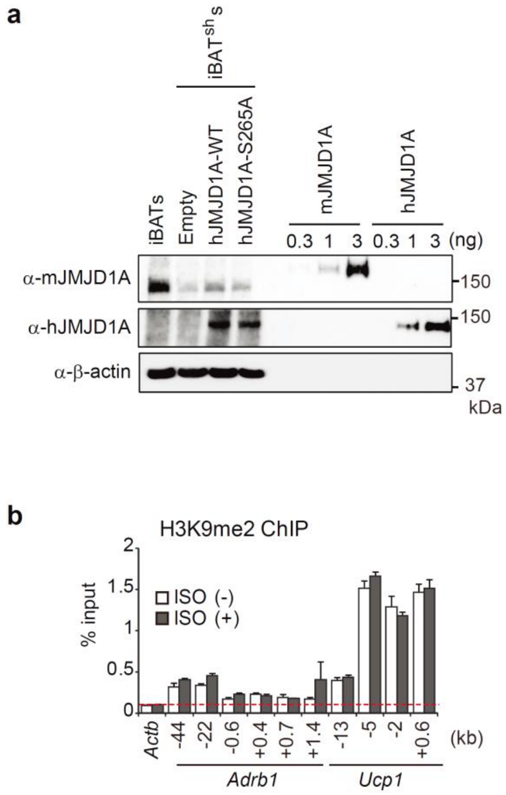

Supplementary Figure 3 | Equivalent protein expressions of overexpressed human JMJD1A and native JMJD1A protein expressions in immortalized brown adipocytes. (**a**) Aliquots of whole cell lysate (60 µg protein) prepared from iBATs, iBAT^{sh}s overexpressing wild-type or S265A human JMJD1A (WT- or S265A-hJMJD1A-iBAT^{sh}s, respectively), or control Zeo^{r}-empty (Empty (Zeo^{r})) along with the indicated amounts of recombinant purified human JMJD1A (full length) or His-tagged mouse JMJD1A (full length) proteins were subjected to SDS-PAGE and immunoblot analysis with anti-mJMJD1A (IgG-F0618) or anti-hJMJD1A (IgG-F1628). The result showed that exogenous human JMJD1A was expressed in iBAT^{sh}s similar level to native JMJD1A in iBATs. Uncropped images of the blots are shown in Supplementary Fig. 14. (**b**) H3K9me2 ChIP-qPCR performed in iBATs at day 8 of differentiation treated with either ISO (1 µM) or vehicle for 2 hr. ChIP-qPCR was performed using the set of primers that amplify the indicated regions from TSS of *Adrb1* or *Ucp1* as listed in Supplementary Table 2. Data were presented as percentage of inputs (± SEM) of three technical replicates. The experiments were performed at least three times with similar results.

a Identified peptides from LC/MS/MS

BRG1
(R)DSEAGSSTPTTSTR(S)
(R)DTALETALNAK(A)
(K)EVDYSDSLTEK(Q)
(R)GLDPVEILQER(E)
(K)GVLLTDGSEK(D)
(R)IGQQNEVR(V)
(R)LLLTGTPLQNK(L)
(R)LMAEDEEGYRK(L)
(R)LTCEEEEEK(M)
(R)QLSEVFIQLPSR(K)
(K)TLMNTIMQLR(K)
(K)AIEEGTLEEIEEEVR(Q)
(K)AVATYHANTER(E)
(K)CDMSALQR(V)
(K)ELPEYYELIR(K)
(R)GLQSYYAVAHAVTER(V)
(R)HEEEFDLFMR(M)
(K)HQEYLNSILQHAK(D)
(K)ILTGTDAPK(A)
(K)IPDPDSDDVSEVDAR(H)
(K)KAENAEGQTPAIGPDGEPLDETSQMSDLPVK(V)
(K)KIPDPDSDDVSEVDAR(H)
(R)LCTVNSVEEK(I)
(R)LMAEDEEGYR(K)
(K)LTQVLNTHYVAPR(R)
(K)QDVDDEYGVSQALAR(G)
(K)VIHVESGK(I)
(K)VIQAGMFDQK(S)
(K)YMIVDEGHR(M)

DNA-directed RNA polymerase II subunit RPB1
(K)DVLSNAHIQNELER(E)
(K)LGGLMDPR(Q)
(K)LTMEQIAEK(I)
(R)MSVTEGGIK(Y)
(K)QDVIEVIEK(A)
(K)SLSEYNNFK(S)
(K)SMESVMVK(Y)
(K)YSPTSPTYSPTSPVYTPTSPK(Y)
(R)YTPQSPTYTPSSPSYSPSSPSYSPTSPK(Y)
(R)AEIQELAMVPR(M)
(K)AHNNELEPTPGNTLR(Q)
(K)AKQDVIEVIEK(A)
(K)FGVEQPEGDEDLTK(E)
(K)HVNEDSQEK(K)
(K)IIITEDGEFK(A)
(R)KLTMEQIAEK(I)
(K)LLVDSNNPK(I)
(R)NSINQVVQLR(Y)
(R)QTFENQVNR(I)
(R)TVITPDPNLSIDQVGVPR(S)
(R)VQFGVLSPDELKR(M)
(K)YSPTSPTYSPTSPK(Y)

ARID1A
(M)AAQVAPAAASSLGNPPPPPSELKK(A)
(K)AGPPVPASHIAPTPVQPPMIR(R)
(K)AMGMTNLPAVGR(K)
(R)CVCVSNTIR(S)
(R)DITFPPGSVEATQPVLK(Q)
(R)ELATNLNVGTSSSAASSLK(K)
(R)ELATNLNVGTSSSAASSLKK(Q)
(R)EMAVVLLANLAQGDSLAAR(A)
(K)EYEVGDPGQR(T)
(R)FGAMGGGGPSAAGGGTPQPTATPTLNQLLTSPSSAR(G)
(K)FPFGISPAQSHR(N)
(R)FPPPQELSQDSFGSQASSAPSMTSSK(G)
(R)GEDPPPDIFAAADSK(K)
(R)GPSPSPVGSPASVAQSR(S)
(R)GYQGYPGGDYGGGPQDGGAGK(G)
(K)HLTTVEGTPGTTEQEGPPPDGLPEK(R)
(K)IERGEDPPPDIFAAADSK(K)
(K)LEEEEEEGVGNDEEMAFLGK(D)
(R)LVLETLSK(L)
(K)LYELGGEPER(K)
(K)LYELGGEPERK(M)
(R)MSYEPNKDPYGSMR(K)
(R)NDMTYNYANR(Q)
(R)NDPFVVDCSDK(L)
(R)NPQMPQYTSPQPGSALSPR(Q)
(R)NSMTPNPGYQPSMNTSDMMGR(M)
(R)QHYPYGGPYDR(V)
(R)QNTGSATQGPAYHGVNR(T)
(R)RDITFPPGSVEATQPVLK(Q)
(K)RPMDGTYGPPAK(R)
(R)SHHAPMSPGSSGGGGQPLAR(T)
(R)SLSFVPGNDFEMSK(H)
(R)SNSVGIQDAFPDGSDPTFQK(R)
(R)SSTLTDEGAK(S)
(R)TDEMLHTDQR(A)
(R)TPQSSSPMDQMGK(M)
(K)VDENHSEFTLYESR(L)
(R)AAGPGLGSVAMGPR(Q)
(R)EEAGGEAAAAAAER(G)
(K)EIGGLTQVNK(N)
(R)ITATMDDMLSTR(S)

BAF60b
(K)ADGDNAGTAGTPGGTPAADK(V)
(K)AQMSNFLASTTNQQEIASLDVK(I)
(R)ELVPESQAYMDLLAFER(K)
(K)IITDVIGNPEEERR(A)
(R)MPTTQETDGFQVK(R)

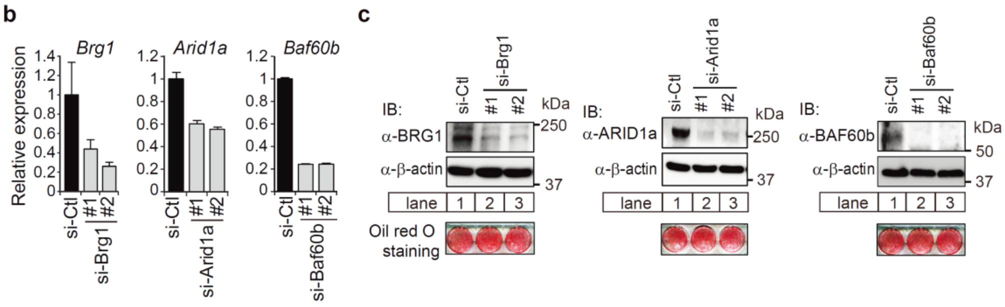

Supplementary Figure 4 | Identification of P-JMJD1A binding proteins. (**a**) Identified peptides for BRG1, DNA-directed RNA polymerase II subunit RPB1 (POLR2A), ARID1A, and BAF60b from LC/MS/MS. (**b**,**c**) Knockdown efficiency of *Brg1*, *Arid1a*, or *Baf60b* in differentiated iBATs were determined by RT-qPCR (**b**) and immunoblot analysis (**c**). Data in (**b**) were presented as fold change relative to control siRNA transfected WT-hJMJD1A iBAT^{sh}s (± SEM) of three technical replicates. Oil Red O staining was performed in the indicated cells (**c**). Uncropped images of the blots (**c**) are shown in Supplementary Fig. 14.

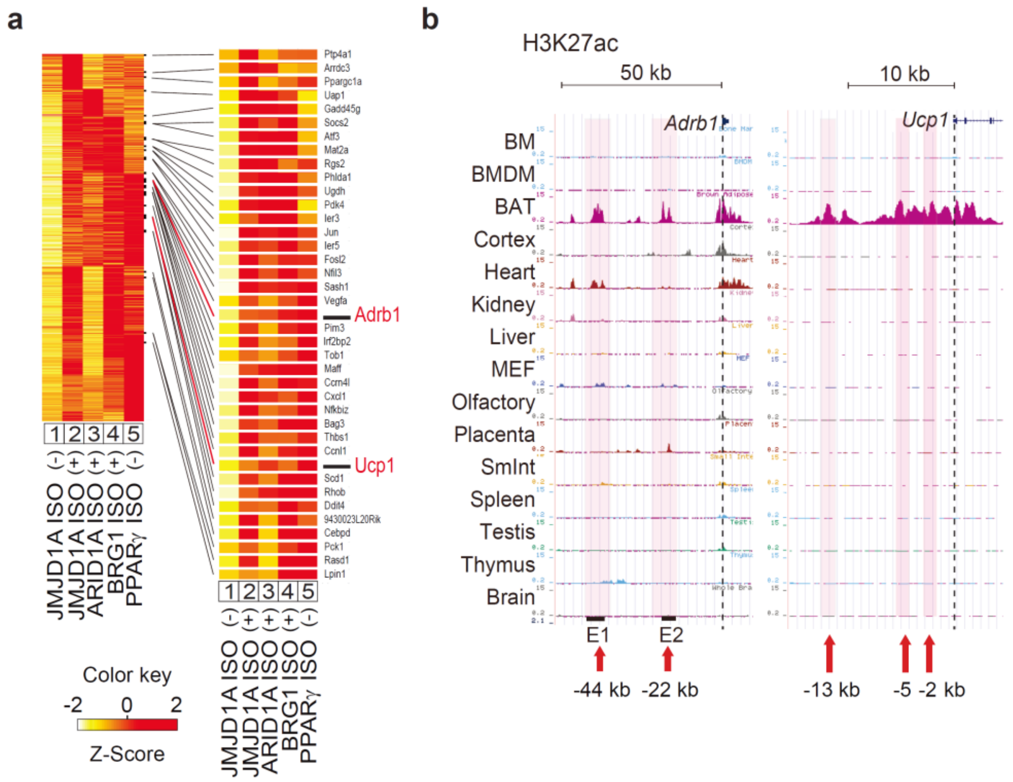

Supplementary Figure 5 | Co-localization of JMJD1A, SWI/SNF, and PPARγ across JMJD1A target genomic regions. (**a**) Heat map showing co-recruitment of ARID1A, BRG1 and PPARγ to ISO-induced JMJD1A binding sites. ISO-induced JMJD1A binding sites were further classified by the Z-scores of ARID1A, BRG1, and PPARγ binding to ISO induced JMJD1A binding sites as described in detail under the Methods. Z-scores of ARID1A, BRG1, and PPARγ bindings were scored based on the number of sequence tags obtained by ChIP-seq of ARID1A, BRG1, and PPARγ. Magnified heat map on the right represents P-JMJD1A target 39 genes shown in Fig. 1**d**. For reference, a color intensity scale is included. (**b**) H3K27ac marks distal to *Adrb1* and *Ucp1* in brown adipose tissue. ChIP-seq profiles of H3K27ac from the indicated normal mouse tissues were obtained from ENCODE/LICR histone modification data in the UCSC genome browser (NCBI37/mm9 assembly). Abbreviations: BM, bone marrow; BMDM, BM-derived macrophages; SmInt, small intestine.

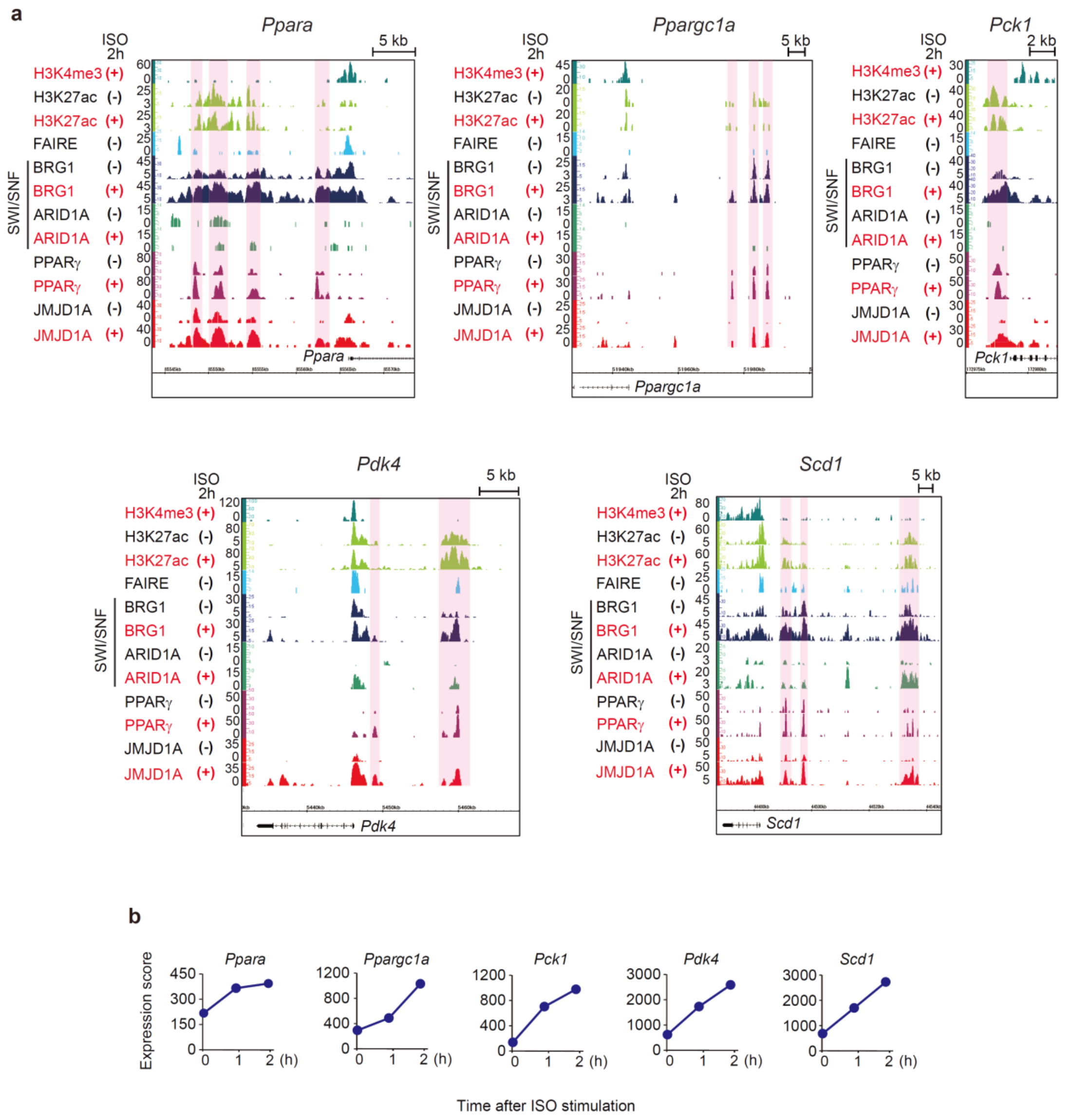

Supplementary Figure 6 | Co-localization of JMJD1A, SWI/SNF, and PPARγ and their target gene expressions. (**a**) ChIP-seq profiles for H3K4me3, H3K27ac, BRG1, ARID1A, PPARγ and JMJD1A and FAIRE-seq open chromatin profile on *Ppara*, *Ppargc1a*, *Pck1*, *Pdk4* and *Scd1* genomic regions. iBATs (day 8) were treated with 1 µM ISO or vehicle for 2 hr and subjected to ChIP-seq or FAIRE-seq analysis. Light pink shadows highlight the enhancers from H3K27ac ChIP-seq data. JMJD1A, SWI/SNF components (ARID1A and BRG1), and PPARγ co-localized at distal enhances of *Ppara*, *Ppargc1a*, *Pck1*, *Pdk4* and *Scd1*. (**b**) mRNA levels of *Ppara*, *Ppargc1a*, *Pck1*, *Pdk4* and *Scd1* in iBATs (day 8) treated with 1 µM ISO for 0, 1, 2 hr were determined by transcriptional microarray analysis.

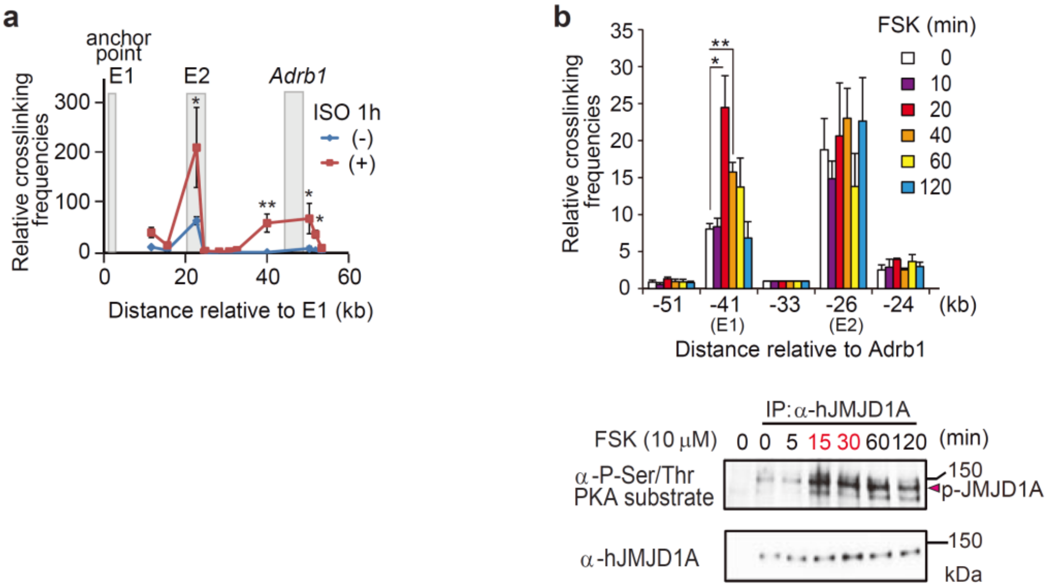

Supplementary Figure 7 | P-JMJD1A mediates PKA-induced enhancer-promoter interaction at the *Adrb1* locus. (**a**) 3C-qPCR analysis of the interaction frequency of the restriction fragments with the indicated anchor points fixed near the enhancer E1. The gray shadows highlight the regions containing E1 and E2 enhancer elements and anchor points. Crosslinked chromatin samples prepared from differentiated iBATs (day 8) were treated with or without ISO (1 µM) for 1 hr, the same was used in Figure 6**a**. Relative interacting frequencies between the indicated regions and the anchor point were measured by qPCR. All PCR signals were normalized to digested/re-ligated bacterial artificial chromosome (BAC) templates. Error bars represent ± SEM of three independent experiments. Student's *t*-test was performed and *P < 0.05 and **P < 0.01 were considered statistically significant. (**b**) Time-course of FSK-induced interactions between *Adrb1* promoter and two distal enhancer regions (-41 kb and -26 kb) detected by 3C. Crosslinked chromatin samples were prepared from iBATs treated with FSK (20 µM) for indicated time. Error bars represent ± SEM from 3 independent experiments. ANOVA was performed followed by Tukey's post hoc comparison and *P < 0.05 and **P < 0.01 were considered statistically significant. Time course of FSK-induced JMJD1A phosphorylation was determined by immunoprecipitation followed by immunoblot analysis (bottom panel). Uncropped images of the blots are shown in Supplementary Fig. 14.

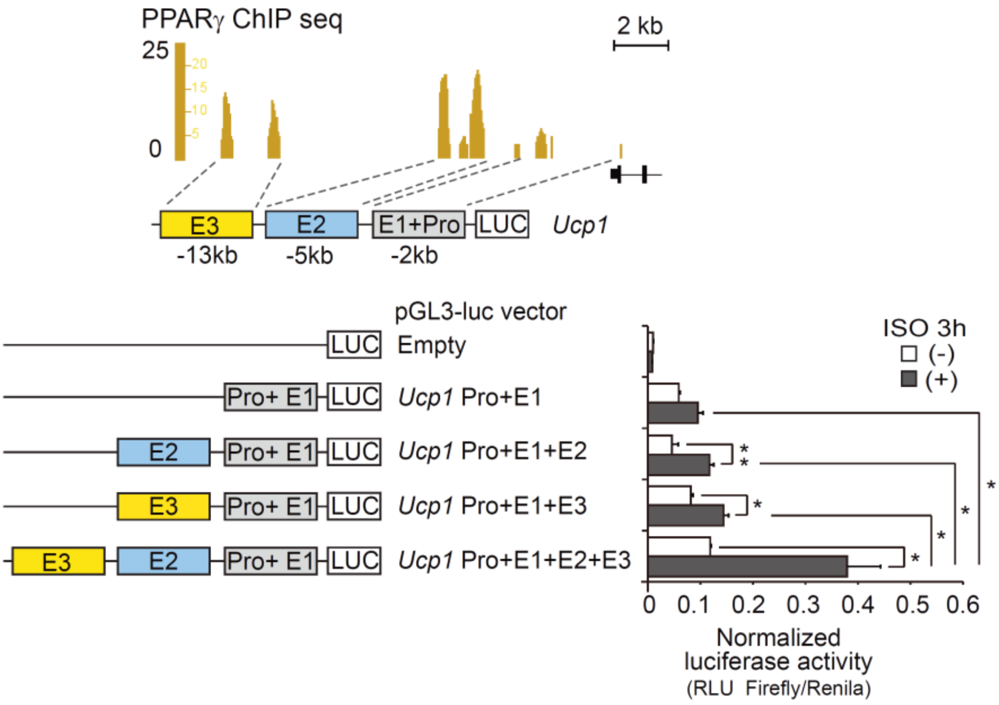

Supplementary Figure 8 | Combination of three *Ucp1* enhancer elements enhances luciferase gene expression. Luciferase reporter activity driven by *Ucp1* promoter and three enhancer elements (E1, E2, and E3). iBATs were transfected with the indicated luciferase reporter plasmids (bottom left panel) and cultured followed by luciferase activity measurement (bottom right panel). Transfected iBATs were incubated with differentiation medium containing 5 μg mL^{-1} insulin and 125 μM indomethacin for 4 days. Isoproterenol (ISO; 10 μM) was added for 3 hr prior to the luciferase reporter assay (bottom right panel). Data were normalized to Renilla internal control. Error bars represent mean ± SEM of three independent experiments. ANOVA was performed followed by Tukey's post hoc comparison and $*P < 0.05$ and $**P < 0.01$ were considered statistically significant.

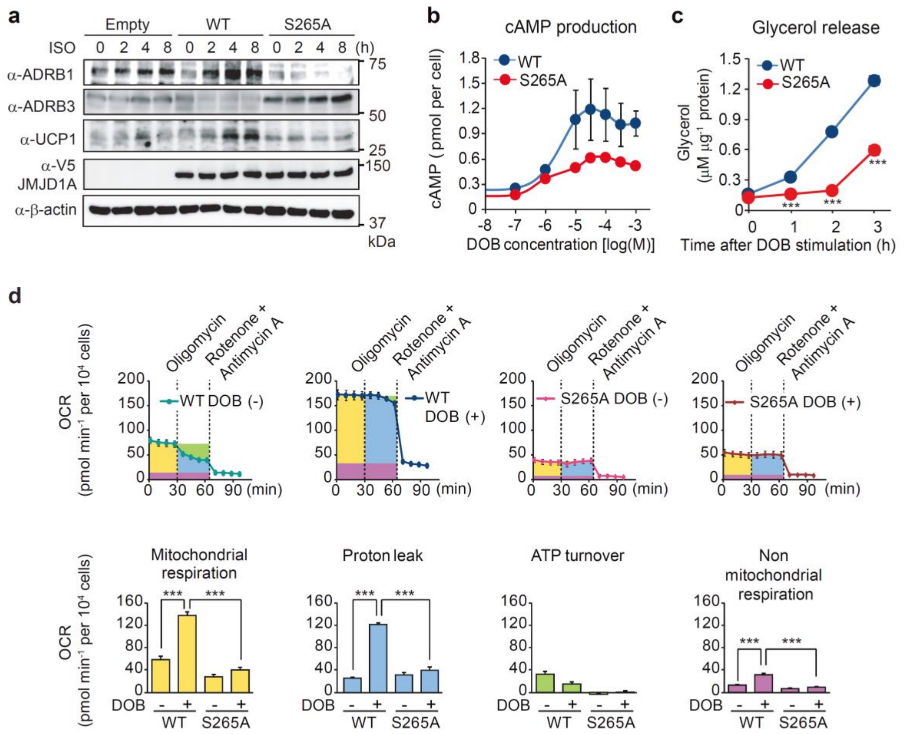

Supplementary Figure 9 | P-S265-JMJD1A mediates β-adrenergic induced thermogenesis. (**a**) Immunoblot analysis for ADRB1, ADRB3 and UCP1 proteins treated with 10 μM ISO for the indicated hours in empty, WT- and S265A-hJMJD1A iBAT^{sh}s. Equal loading of the proteins was confirmed by β-actin. Uncropped images of the blots are shown in Supplementary Fig. 15. (**b**) Cyclic AMP productions by dobutamine (DOB) treatment (5 min) in WT- or S265A-hJMJD1A iBAT^{sh}s at day 4 of differentiation. The experiments were performed for three times with similar results and the representative one is shown. Error bars represent the range of duplicate experiments. (**c**) Dobutamine-induced glycerol release in WT- and S265A-hJMJD1A iBAT^{sh}s treated with dobutamine (DOB) for the indicated hours. Data are mean ± SEM of three independent experiments (error bars are too tiny to see). Student's *t*-test was performed and ***$P < 0.001$ was considered statistically significant. (**d**) The metabolic profile of iBAT^{sh}s expressing hJMJD1A (day 8) was assessed using a Seahorse XF24 extracellular Flux analyzer. A representative curve of the OCRs of WT- or S265A- hJMJD1A expressing iBAT^{sh}s in their basal states and on treatment with drugs used to dissect the multiple components of the respiration process is plotted in the top left panel. The parameters analyzed on the same plate are represented by different colors in the upper panels and quantitated in other panels. Mitochondrial respiration (bottom left panel) and proton leak levels (bottom second left panel) in WT- and S265A-hJMJD1A iBAT^{sh}s (day 8) before and after stimulation with dobutamine (DOB). Data are presented as

percentage of total basal respiration. Data are mean ± SEM of five technical replicates. ANOVA was performed followed by Tukey's post hoc comparison and ***$P < 0.001$ was considered statistically significant.

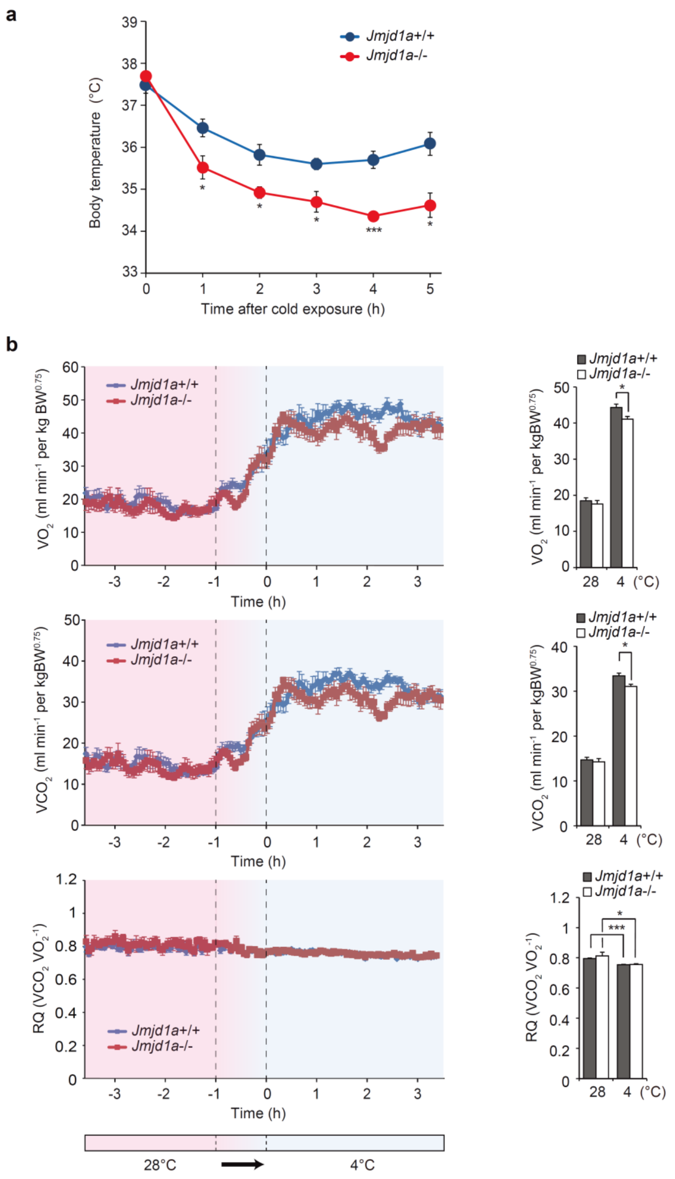

Supplementary Figure 10 | *Jmjd1a*-/- mice are cold intolerant and exhibit decreased oxygen consumption compared to *Jmjd1a*+/+ mice under cold exposure. (**a**) Core rectal temperature of *Jmjd1a*+/+ and *Jmjd1a*-/- mice at indicated time points during exposure to 4°C were measured as described 8 (6-11 weeks of age, n = 5). Mice were acclimated to thermoneutrality for 24 hr prior to experiments. Student's *t*-test was performed and **P* < 0.05 and ****P* < 0.001 were considered statistically significant. (**b**) Oxygen consumption (VO_2), CO_2 production (VCO_2) and respiratory quotient (RQ) measured by indirect calorimetry in individually placed *Jmjd1a*+/+ and *Jmjd1a*-/- mice. The mice acclimated to thermoneutrality for 1-2 weeks were placed at 28°C in laboratory incubator and shifted to 4°C taking 1 hr (6-8 weeks of age, n = 6 mice per

group). Dotted lines indicate finishing of 28°C and starting of 4°C. Average of VO_2, VCO_2 and RQ from 6 mice measured during the entire 28°C and 4°C periods are calculated (right panels). Error bars represent ± SEM. Student's *t*-test was performed and $*P < 0.05$ and $***P < 0.001$ were considered statistically significant.

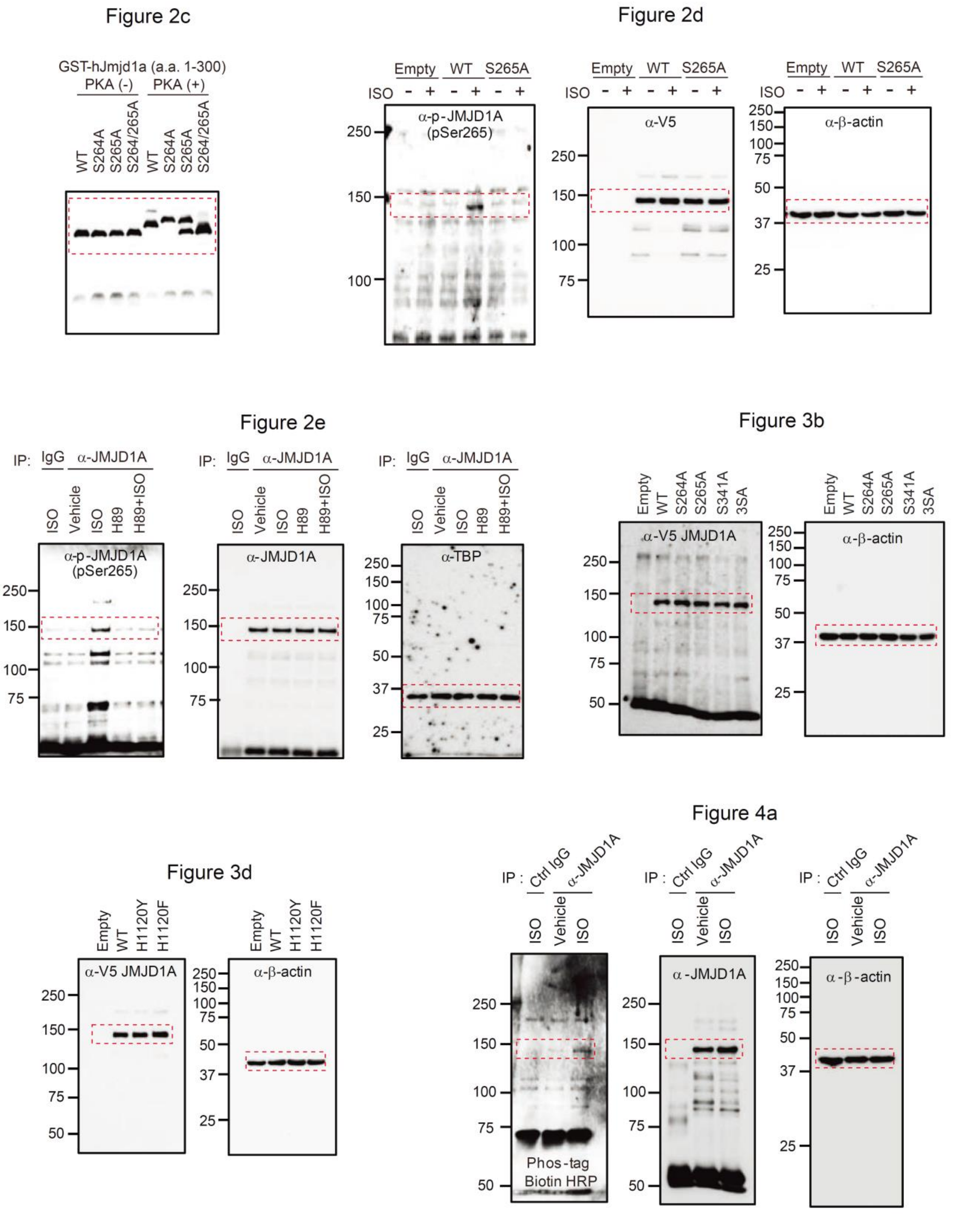

Supplementary Figure 11 | Representative original images of immunoblot analysis for Figs. 2, 3, 4a.

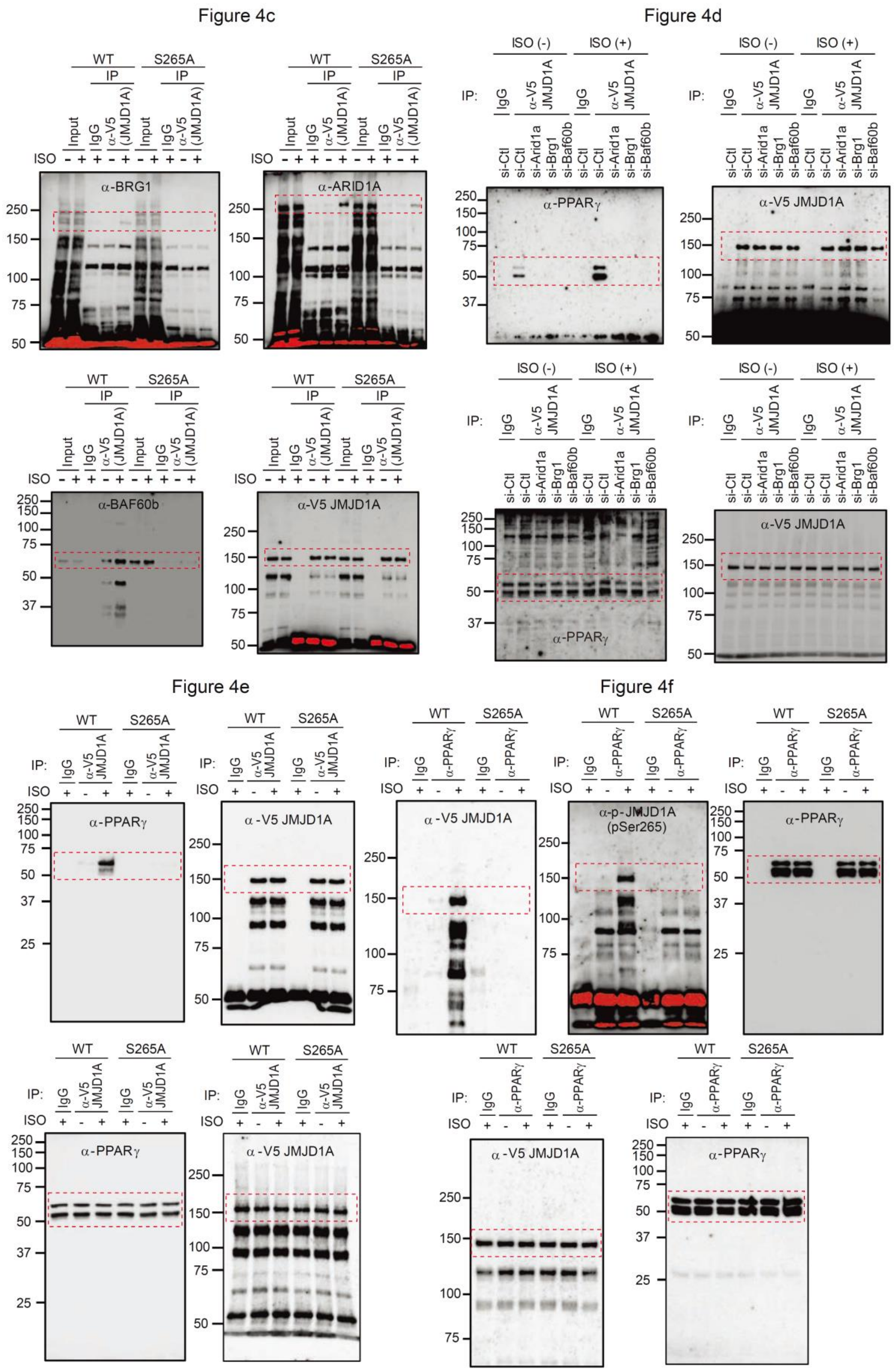

Supplementary Figure 12 | Representative original images of immunoblot analysis for Fig. 4.

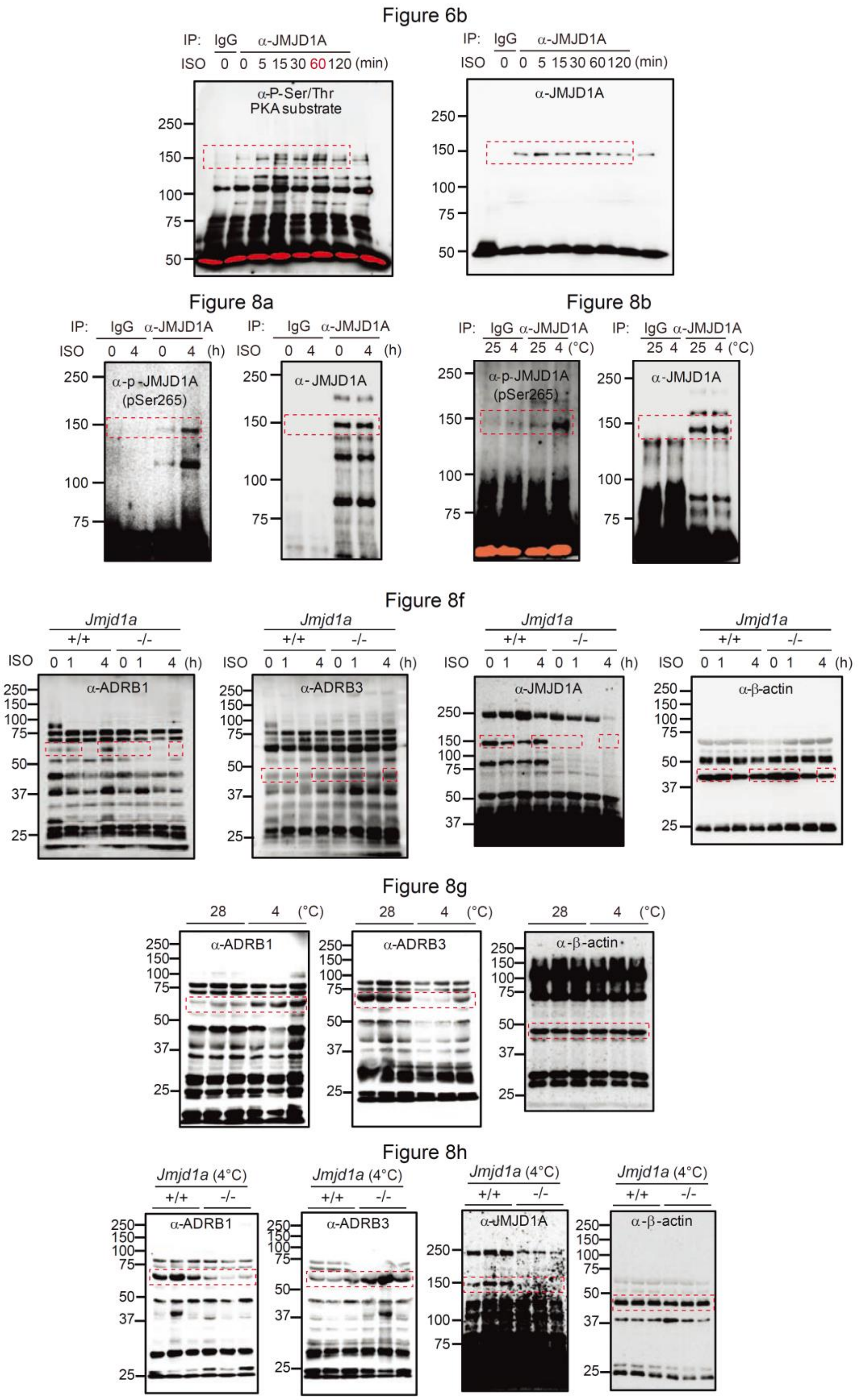

Supplementary Figure 13 | Representative original images of immunoblot analysis for Figs. 6, 8.

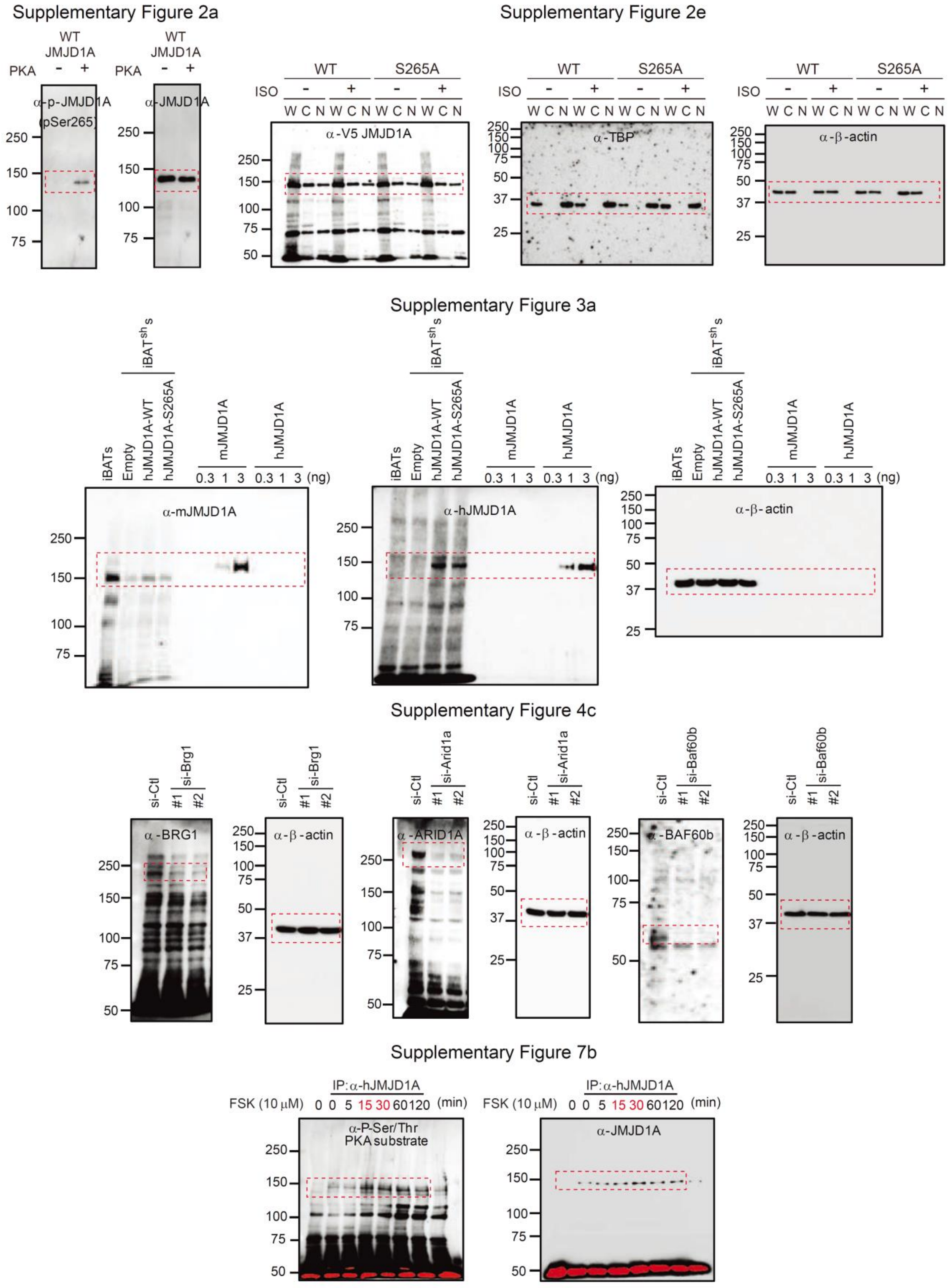

Supplementary Figure 14 | Representative original images of immunoblot analysis for Supplementary Figs. 2-4, 7.

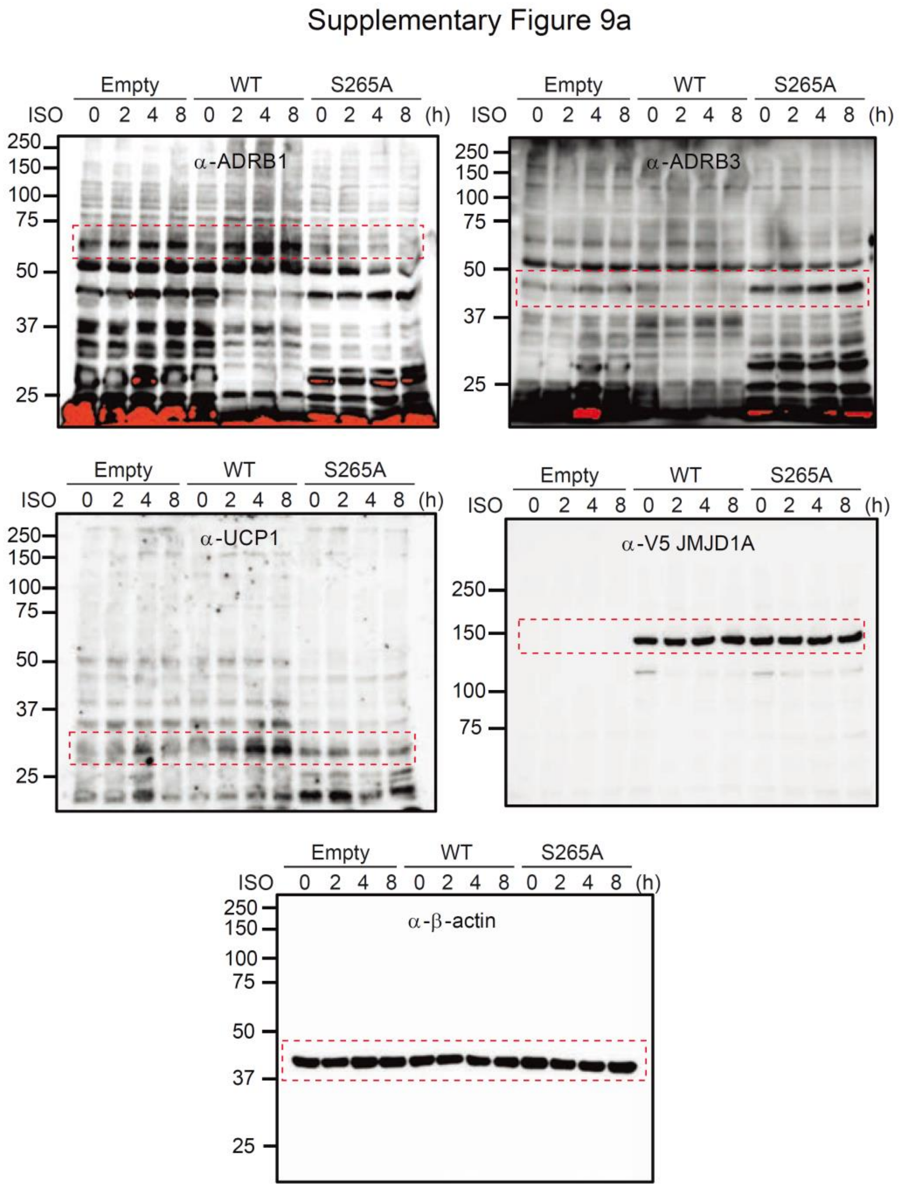

Supplementary Figure 15 | Representative original images of immunoblot analysis for Supplementary Fig. 9.

Supplementary Table 1. Antibodies

Antibody		Source	Catalog No /Clone No	Dilutions or concentrations
anti-human JMJD1A	Monoclonal	Our laboratory	IgG-F0026	10 μg mL^{-1} for IP
anti-human JMJD1A	Monoclonal	Our laboratory	IgG-F1628	5 μg mL^{-1} for IB
anti-mouse JMJD1A	Monoclonal	Our laboratory	IgG-F0231	50 μg mL^{-1} for ChIP (together with 50 μg mL^{-1} of IgG-F0618)
anti-mouse JMJD1A	Monoclonal	Our laboratory	IgG-F0618	5 μg mL^{-1} for IB,<br>10 μg mL^{-1} for IP
anti-P-JMJD1A (pSer265)	Polyclonal	Our laboratory	#11890-2	1:1000 for IB
anti-H3K4me3	Monoclonal	Dr. Kimura	CMA304	10 μg mL^{-1} for ChIP
anti-H3K9me2	Monoclonal	Dr. Kimura	6D11	10 μg mL^{-1} for ChIP
anti-H3K27ac	Monoclonal	Dr. Kimura	CMA309	10 μg mL^{-1} for ChIP
anti-H3Ac	Polyclonal	Millipore	#06-599	1:100 for ChIP
anti-Pol II	Monoclonal	Dr. Kimura	C1389	20 μg mL^{-1} for ChIP
anti-ADRB1	Polyclonal	Santa Cruz	sc-568 (V-19)	1:1000 for IB
anti-ADRB3	Polyclonal	Santa Cruz	sc-50436 (M-50)	1:1000 for IB
anti-UCP1	Monoclonal	R&D Systems	MAB6158	1 μg mL^{-1} for IB
anti-BRG1	Polyclonal	Cell Signaling	#3514	1:1000 for IB
anti-BRG1	Monoclonal	Abcam	ab110641	1 μg mL^{-1} for ChIP
anti-ARID1A /BAF250A	Monoclonal	Cell Signaling	#12354	1:1000 for IB
anti-ARID1A	Monoclonal	Millipore	#04-080	10 μg mL^{-1} for ChIP
anti-BAF60B /SMARCD2	Polyclonal	Santa Cruz	sc-132105 (K-12)	1:1000 for IB
anti-PPARγ	Monoclonal	Santa Cruz	sc-7273 (E-8)	4 μg mL^{-1} for ChIP (together with 4 μg mL^{-1} of IgG-A3409)

anti-PPARγ	Monoclonal	Our laboratory	IgG-A3409	0.5 μg mL^{-1} for IB
anti-C/EBPα	Polyclonal	Santa Cruz	sc-61X	20 μg mL^{-1} for ChIP
anti-C/EBPβ	Polyclonal	Santa Cruz	sc-150X	20 μg mL^{-1} for ChIP
anti-C/EBPδ	Polyclonal	Santa Cruz	sc-636X	20 μg mL^{-1} for ChIP
anti-P-Ser/Thr PKA substrate	Polyclonal	Cell Signaling	#9621	1:1000 for IB
anti-GST	Monoclonal	Cell Signaling	#2624	1:1000 for IB
anti-V5-tag	Monoclonal	Invitrogen	R960-25	1 μg mL^{-1} for IB,<br>10 μg mL^{-1} for IP, IF
anti-β-actin	Monoclonal	Sigma	A5441	1:5000 for IB
anti-TBP	Monoclonal	Novus Biologicals	NB500-700	1.7 μg mL^{-1} for IB

Supplementary Table 2. ChIP-qPCR primers

Gene	Sequence		Amplified regions
	Forward Primer	Reverse Primer	
Adrb1	5'-CTCCTGGGCGAAAGTGAGA-3'	5'-CCTGGTGACGGTTGGAGGT-3'	*Adrb1 E1* (-44 kb)
	5'-GGGGACCAACACCACCTAATC-3'	5'-TGAGAAGCCACGGACGTATG-3'	*Adrb1 E2* (-22 kb)
	5'-CGTCTCCGAGACGCCTGTAA-3'	5'-GCAAGAGCGCCCTTATTTCC-3'	*Adrb1* (-0.6 kb)
	5'-TGGGAGTACGGCTCCTTCTT-3'	5'-GCGATGACACACAGGGTCTC-3'	*Adrb1* (+0.4 kb)
	5'-TGCTACAACGACCCCAAGTG-3'	5'-CACGTAGAAGGAGACGACGG-3'	*Adrb1* (+0.7 kb)
	5'-CCCTAGAGGAAGAGAGCCCG-3'	5'-GGCTTTGCATTCACCTGCTT-3'	*Adrb1* (+1.4 kb)
Ucp1	5'-GCAACCCTCTCCCATCAGTG-3'	5'-GCCTAACACCGTGCTTCTCA-3'	*Ucp1* (-13 kb)
	5'-TGCAACCCCTCACCTTTTAC-3'	5'-CTCCTTCCATCATCCCTTCA-3'	*Ucp1* (-5 kb)
	5'-TCACCCTTGACCACACTGAA-3'	5'-GTGAGGCTGATATCCCCAGA-3'	*Ucp1* (-2 kb)
	5'-GCCAGGCCTGTAAACTCTGA-3'	5'-TGGGTTAAAAATGGCCTCTG-3'	*Ucp1* (+0.6 kb)
Actb	5'-TGAGGTACTAGCCACGAGAGAG-3'	5'-ACACCCGCCACCAGGTAAGCA-3'	*Actb*
Ppib	5'-CTCACCCCAACTAGTCTAATCC-3'	5'-GTGACACACAGTGACTAACTTCC-3'	*Cyclo*

Supplementary Table 3. RT- qPCR primers

Gene	Sequence	
	Forward Primer	Reverse Primer
Brg1	5'-TCCCCAAACCCACCTAACCT-3'	5'-CTGACGTCCACTGCTGTCTTT-3'
Arid1a	5'-CCCCTCAGTCATCCAGTCCA-3'	5'-GGTCCCTGTTGTTGCGAGTA-3'
Adrb1	5'-GCTGGGAGTACGGCTCCTT-3'	5'-GCCGTCACACACAGCACAT-3'
Baf60b	5'-ATCTGGTGGAGTGGCATCG-3'	5'-AGGGTGCACTTGACATTGAGA-3'
Ucp1	5'-AAGCTGTGCGATGTCCATGT-3'	5'-AAGCCACAAACCCTTTGAAAA-3'
Ppargc1a	5'-AACCACACCCACAGGATCAGA-3'	5'-TCTTCGCTTTATTGCTCCATGA-3'
Ppib	5'-GGAGATGGCACAGGAGGAA-3'	5'-GCCCGTAGTGCTTCAGCTT-3'
Pdk4	5'-CAAAGACGGGAAACCCAAGC-3'	5'-CGCAGAGCATCTTTGCACAC-3'
Hk1	5'-AGAAAGGCTTCCTCTTCCGG-3'	5'-CGCTAATCGGTCACTCTCGA-3'
Pck1	5'-CACCATCACCTCCTGGAAGA-3'	5'-GGGTGCAGAATCTCGAGTTG-3'

Supplementary Table 4. 3C-qPCR primers

Adrb1 anchor	Primer sequence	
Forward primer	5'-GATGTTCTAGGTGGGCAGGA-3'	
Reverse primers (kb from the anchor)	-100	5'-GGTAAGGAGGGATGGAGAGG-3'
	-51	5'-GGGATGTCCTTTATGCAGGT-3'
	-43	5'-CAGTTTTCCACCCCTGTCAC-3'
	-41	5'-GGGTTTATCCCACTGCAGAA-3'
	-33	5'-CTCTGGCGAGAAGCTTGTTT-3'
	-26	5'-GCCCACATGCTTTGAGAAAT-3'
	-24	5'-GAGACTTCGGGAGGAAAAGC-3'
	-21	5'-AGTTGTCAACAGGGCAAAGC-3'
TaqMan® MGB probe	(6-FAM) 5'-TTCACCCAGTCTGTTTATCCAA-3' (MGB)	

Enhancer E1 anchor	Primer sequence	
Forward primer	5'-CCTGTTCACCCAGCTTAAATG-3'	
Reverse primers (kb from the anchor)	12	5'-TAAACCAACAGCAGGCTTCC-3'
	15	5'-TGACCTTTGTGGAAAGCACTC-3'
	23	5'-GGGGTGGGGATGTCCTTTAT-3'
	24	5'-CACCTCTTTGCCTAGCTCTCA-3'
	25	5'-GAGACTTCGGGAGGAA-3'
	28	5'-GTTGTCAACAGGGCAAAGC-3'
	31	5'-GGTCCATTTCTTGCCTCTGA-3'
	33	5'-TGAGCTAGAAAATCAACTGACAGG-3'
	49	5'-GACCCTGAGCCAAAACCATA-3'
	51	5'-GTCCGGCTTGTCAGAGACAT-3'
	52	5'-TTGACCAGGTTTTCCTCTCC-3'
TaqMan® MGB probe	(6-FAM) 5'-TGCTGAGGAGACGGCTTAGT-3' (MGB)	

Supplementary Methods

Retroviral vectors and infection

To construct a retroviral vector for JMJD1A (pMXs-hJMJD1A-V5-IRES/Zeo), we subcloned DNA sequence encoding human JMJD1A open reading frame (ORF) into an expression vector pMX (a kind gift from Dr. Toshio Kitamura, The University of Tokyo) driven by LTR promoter, in which the original puromycin-resistant sequences were replaced by Zeocin™-resistant sequences. Mutant versions of JMJD1A: S264A, S265A, S341A, 3SA, H1120Y, or H1120F were generated by PCR-based site-directed mutagenesis. To construct retroviral vector for shRNA targeting Jmjd1a, forward primer 5'-gatctttgccgatgacctttcagataattcaagagattatctgaaaggtcatcggttttgttcc-3' and reverse primer 5'-agctggaacaaaaccgatgacctttcagataatctcttgaattatctgaaaggtcatcggcaaa-3' were annealed and subcloned into a mouse U6 promoter-driven expression vector pRetro/Puro-Super3 (a kind gift from Dr. T. Kitamura) in which puromycin resistant marker sequences were replaced by neomycin resistant marker sequences. Retroviruses were produced by transfecting each plasmid into Platinum-E (Plat E) packaging cells (a kind gift from Dr. T. Kitamura) using GeneJuice (Novagen) as described previously [1, 2].

Purification of GST-fusion peptide and in vitro PKA assay

GST-fusion JMJD1A proteins (a.a. 1-300: WT, S264A mutant, S265A mutant, and S264A/S265A double mutant) were produced in *E.coli*, purified using glutathione-Sepharose 4B (GE Healthcare), and eluted with reduced glutathione buffer (50 mM Tris-HCl, pH 8.0, 10 mM reduced L-glutathione) as described previously [3, 4]. To perform in vitro kinase assay, 1 μg of GST-fusion WT, S264A, S265A, or S264A/S265A JMJD1A (a.a. 1-300) was incubated with protein kinase A (2,500 U per mg protein) (New England Biolabs) in kinase assay buffer (20 mM Tris-HCl, pH 7.5, 1 mM $MgCl_2$) containing 2.5 mM ATP for 1 hr at 30°C, and terminated by addition of SDS sample buffer (30 mM Tris-HCl, pH 6.8, 3% SDS, 5% glycerol, 0.004% bromophenol blue, 2.5% 2-mercaptoethanol). Phosphorylation of JMJD1A proteins were resolved in Phos-tag SDS-PAGE gels (Wako) followed by immunoblotting with anti-GST antibody.

Plasmid constructions

The sequence of human JMJD1A open reading frame (ORF) was amplified by PCR using cDNA from HUVEC cells as a template and cloned into a Gateway® entry vector (pENTR-SD/D-hJMJD1A) (Invitrogen). The cytomegalovirus (CMV) promoter-driven expression vector carrying human JMJD1A genes (pcDNA3.2-hJMJD1A-V5) were generated by transferring JMJD1A ORF into pcDNA3.2-V5-DEST using Gateway® cloning technology (Invitrogen). To create the mouse *Adrb1*-luciferase reporter plasmids, the intergenic sequences from either promoter region (up to -2 kb from TSS) (chr19:56794862-56796861), E1 (chr19:56752006-56753567) (-43 to -45 kb region from TSS), or E2 (-20 to -21 kb region from TSS) (chr19:56775190-56776718) were amplified by PCR using mouse genomic DNA from C57BL/6J mouse as a template, and cloned into pGL3-basic vector (Promega). The resulting constructs were designated pGL3-m*Adrb1*-pro, pGL3-m*Adrb*-pro-E1, pGL3-m*Adrb1*-pro-E2 and pGL3-m*Adrb1*-pro-E1-E2, respectively. Mutations were introduced into pGL3-m*Adrb*1-pro-E1 constructs by site-directed mutagenesis (pGL3-m*Adrb1*-pro-mut E1) using a KOD mutagenesis kit (TOYOBO) according to the manufacturer's protocol. Oligonucleotides were designed to mutate PPRE site at region E1: 5'-aagcttccagtgagcaacacgtcact-3' and 5'-actttcgttctcacccctgt-3'. To create the mouse *Ucp1*-luciferase reporter plasmids, the intergenic sequences from either promoter plus enhancer E1 region (up to -3.3 kb from the translation initiation site) (chr8:85,811,258-85,814,509), E2 (-4.3 to -6.3 kb region from the translation initiation site) (chr8:85,808,201-85,810,200), or E3 (-11 to -14 kb region from the translation initiation site) (chr8:85,800,002-85,803,500), were amplified by PCR and cloned into pGL3-basic vector (Promega) as described above. The resulting constructs were designated pGL3-m*Ucp1*-Pro+E1, pGL3-m*Ucp1*-pro+E1+E2, pGL3-m*Ucp1*-pro+E1+E3, and pGL3-m*Ucp1*-pro+E1+E2+E3, respectively. C-terminal deletion bacterial expression vector encoding a.a. 1-300 of human JMJD1A was generated by PCR and inserted into TAC promoter-driven GST fusion pGEX-4T2 (GE Healthcare) (pGEX-4T2-hJMJD1A (1-300)). To create point mutant JMJD1A bacterial expression plasmids pGEX-4T2-hJMJD1A (1-300, S264A), pGEX-4T2-hJMJD1A

(1-300, S265A), and pGEX-4T2-hJMJD1A (1-300, S264A/S265A), DNA sequence encoding S264 and/or S265 were mutated by PCR-based site-directed mutagenesis.

Luciferase reporter assay

iBATs (6 x10^5 cells/24-well) were transfected with the indicated pGL3 reporter (150 ng) and Renilla luciferase plasmids (pRL-CMV) (9 ng) using GeneJuice (Novagen) according to the manufacturer's instructions. Two days after transfection, the cells were harvested and the firefly and Renilla luciferase activities were measured using Dual-Glo Luciferase assay system (Promega). Firefly luciferase signal was normalized to Renilla luciferase signal. All luciferase assay data represent ± SEM of triplicate samples.

Animal experiments

Mice were fed standard chow (CE-2, CLEA Japan Inc.) ad libitum in a temperature controlled environment with 12 hr light/dark cycles. *Jmjd1a+/+* and *Jmjd1a-/-* mice previously described [5] or C57BL/6J mice were evaluated for cold- or ISO-induced changes in chromatin conformation and histone modification by 3C. For 3C assay, brown adipose tissues were taken, minced in cold PBS and cross linked as described under "Chromosome conformation capture (3C) assay". For immunoblot analysis, tissues were flash frozen in liquid nitrogen and stored at -80°C. For the cold-induced changes, mice were placed at 28°C for 4 hr and shifted to 4°C environment for 4 hr. At the end of the experiments, tissues were harvested and snap frozen. Energy expenditure was measured using indirect calorimetry (MK-5000RQ; Muromachi) as described previously [6, 7]. Core body temperature was monitored using a rectal thermometer. 6-11 weeks mice were acclimated to thermoneutrality (28°C) for 24 hr prior to experiments as described [8]. All mice experiments were approved by the Animal Care and Use Committee of the University of Tokyo.

Cyclic AMP assay

Cyclic AMP assay was performed using a Lance® Ultra cAMP kit (PerkinElmer) according to the manufacturer's protocol with slight modifications. Briefly, 6 x10^4 iBAT cells/well were plated into 24-well plate. After 4 days of differentiation, cells were

washed and treated with stimulation buffer (0.1% BSA stabilizer, 0.5 mM IBMX, 5 mM HEPES in Hanks' balanced salt solution (HBSS) medium) containing with serial concentrations of dobutamine (Sigma-Aldrich) for 5 min. Cells were lysed by adding 1% Triton X diluted in HBSS medium by pipetting. Eu-cAMP tracer and ULight-anti-cAMP were serially added to lysed cells and incubated for 1 hr at RT. Fluorescence signal was measured at 665 nm using ARVO X5 microplate reader (PerkinElmer) and interpolated to standard curve to determine the value.

Glycerol release

β-Adrenergic-stimulated glycerol release from iBATs was performed using a Lypolysis Assay Kit (ZenBio) according to the manufacturer's protocol. Briefly, 7.5 $x10^4$ iBAT cells/well were plated into 96-well plate. After 8 days of differentiation, cells were washed and treated with 100 μM dobutamine for 0, 1, 2, or 3 hr at 37°C. The optical density of produced quinoneimine dye was measured at 540 nm.

Flux analysis

Oxygen consumption was measured using a Seahorse XF24 extracellular flux analyzer as described previously [9]. iBATs were induced for differentiation as described above and on day 7, adipocytes were detached with EDTA (0.53 mM, pH 7.4) and re-seeded at 4.0 $x10^4$ cells/well into XF24 V7 cell culture microplates (Seahorse Bioscience). On the next day, the medium was replaced with pre-warmed XF24 assay medium (DMEM, 2 mM glutamine, 1 mM pyruvate and 25 mM glucose) in the presence or absence of 100 μM dobutamine (Sigma) for 1 hr. Following the measurement of dobutamine induced respiration, ATP turnover was evaluated in response to 40 μg mL^{-1} oligomycin (Sigma). Mitochondrial respiration was blocked by adding both 1 μM rotenone (Sigma) and 1 μM antimycin A (Wako) and the residual OCR was considered as non-mitochondrial respiration. Proton leak was calculated by subtracting the ATP turnover and the non-mitochondrial respiration components of total basal and dobutamine induced respiration.

RNA interference

ARID1A, BRG1 and BAF60b were depleted from iBAT cells with the following synthetic small interference RNAs targeting *Arid1a*, *Brg1*, and *Baf60b*: *Arid1a* (5’-ggacccaggacagagaacauuacua-3’ (*Arid1a* #1) or 5’-ggaugacauguugucuacccggucu-3’ (*Arid1a* #2)), *Brg1* (5’-ccagcucccugagaagguagaguau-3’ (*Brg1* #1) or 5’-ccaaacuccgugcaacgaaccauaa-3’ (*Brg1* #2)), and *Baf60b* (5’-gcgaguacaucaacugcaaucguua-3’ (*Baf60b* #1) or 5’-cccagagucucaggcauacauggau-3’ (*Baf60b* #2)). Stealth RNAi Negative control Med GC (Invitrogen) was used for a negative control. iBATs were transfected with each siRNA every other day from the day of spreading using Lipofectamine® RNAi MAX reagent (Invitrogen) following the protocol we previously described [10].

Demethylation activity assay

HTRF-FRET demethylation was assayed using a biotinylated dimethyl-Histone H3K9me2 (1-21) peptide substrate (AnaSpec) and a europium cryptate-labeled anti-unmethylated Histone H3K9 specific monoclonal antibody (anti-H3K9me0-Eu(K)) (Sceti Medical Labo). The recombinant JMJD1A and the substrate were reacted in the enzymatic buffer (50 mM HEPES-NaOH, pH 7.0, 0.01% Tween 20, 50 μM ammonium iron (II) sulfate, 50 μM ascorbic acid and 50 μM α-ketoglutarate) at RT for 30 min. For terminating and measuring JMJD1A catalyzed demethylation, the detection reactions were supplemented with both an anti-H3K9me0-Eu(K) and a XL665-conjugated streptavidin (SA-XL665, Sceti Medical Labo) dissolved in EDTA containing detection buffer (Sceti Medical Labo). Each reaction was incubated for 1 hr at RT before reading on the time resolved-FRET signal using ARVO X5 microplate reader (PerkinElmer). Fluorescence was measured at 620 nm and 665 nm, and the calculated HTRF ratio (665 nm/ 620 nm) was presented as Delta F% (DF%) = ((Sample Ratio – Ratio neg) /Ratio neg) x100. Histone demethylation assay using MALDI-TOF MS was performed as described previously [11]. Briefly, 50 μM histone H3K9 me1, me2 or me3 (a.a. 1-15) was reacted at 37°C for 30 min with 1 μM eXact™-tag purified JMJD1A as described below. Reaction product was analyzed using MALDI-TOF MS (AXIMA Performance, Shimadzu).

Immunofluorescence analysis

iBATs plated on slide glasses were fixed with 4% paraformaldehyde in PBS for 10 min at RT and permeabilized with 0.2% NP-40 in PBS for 20 min. After an incubation step with 10% goat serum (Vector Labs) for 30 min, the cells were stained with 10 μg mL^{-1} of anti-V5 antibody (Invitrogen) at 4°C overnight. Slides were incubated with 6 μg mL^{-1} of Alexa Fluor 488 conjugated AffiniPure goat anti-mouse IgG (Jackson ImmunoResearch) and covered with VECTASHIELD mounting medium containing DAPI (Vector Labs). Immunofluorescence was captured with LEICA DMI 6000B microscope (Leica microsystems).

Baculovirus production and protein purification from Sf9 cells

For purifying human JMJD1A protein, baculovirus expressing eXact™-tagged JMJD1A was produced. To create pFastBac1-eXact-hJMJD1A (WT) and pFastBac1-eXact-hJMJD1A (S265A) plasmids, fragments of eXact™ tag sequence and either WT or S265A hJMJD1A sequence were cloned into pFastBac1 vector, respectively (Invitrogen). Recombinant baculovirus was generated using Bac-to-Bac baculovirus expression system (Invitrogen). Baculovirus expressing eXact™-tagged WT or S265A hJMJD1A was infected to Sf9 cells. The harvested cell pellets were lysed in lysis buffer (50 mM MES-KOH, pH 6.5, 500 mM sodium acetate, 1% NP-40, 5 μg mL^{-1} pepstatin A, 10 μg mL^{-1} leupeptin, 2.8 μg mL^{-1} aprotinin, 1 mM benzamidine, 0.5 mM PMSF, 1 mM TCEP) and their supernatants were incubated with Profinity eXact™ purification resin (Bio-Rad Laboratories). After washing steps, WT or S265A hJMJD1A protein was eluted using elution buffer (50 mM MES-KOH, pH 6.5, 100 mM NaF, 5 μg mL^{-1} pepstatin A, 10 μg mL^{-1} leupeptin, 2.8 μg mL^{-1} aprotinin, 1 mM benzamidine, 0.5 mM PMSF, 1 mM TCEP). For purifying mouse JMJD1A protein, baculovirus expressing N-terminally tagged (6xHis-TEV) JMJD1A was produced. Mouse JMJD1A open reading frame was cloned into pENTR/SD/D-TOPO vector and subsequently transferred to pDEST10 vector (Invitrogen) using the LR clonase (Invitrogen). Recombinant baculovirus was generated and infected to Sf9 cells. The lysed cell pellets in lysis buffer (10 mM HEPES-KOH, pH 7.5, 1.5 mM $MgCl_2$, 10 mM KCl, 1mM EDTA, 1mM EGTA, 5 μg mL^{-1} pepstatin A, 10 μg mL^{-1} leupeptin, 2.8 μg mL^{-1} aprotinin,

1 mM benzamidine, 0.5 mM PMSF, 1 mM TCEP) were applied to His-Gravi trap™ column (GE Healthcare) and were eluted by an imidazole gradient (50-500 mM) in elution buffer (20 mM HEPES-KOH, pH 7.5, 500 mM NaCl, 10% glycerol, 5 μg mL^{-1} pepstatin A, 10 μg mL^{-1} leupeptin, 2.8 μg mL^{-1} aprotinin, 1 mM benzamidine, 0.5 mM PMSF, 1 mM TCEP).

Supplementary References

1. Yamamoto J, *et al.* A Kruppel-like factor KLF15 contributes fasting-induced transcriptional activation of mitochondrial acetyl-CoA synthetase gene AceCS2. *J Biol Chem* 279, 16954-16962 (2004).

2. Watanabe-Okochi N, *et al.* AML1 mutations induced MDS and MDS/AML in a mouse BMT model. *Blood* 111, 4297-4308 (2008).

3. Sumi K, *et al.* Cooperative interaction between hepatocyte nuclear factor 4 alpha and GATA transcription factors regulates ATP-binding cassette sterol transporters ABCG5 and ABCG8. *Mol Cell Biol* 27, 4248-4260 (2007).

4. Ohguchi H, *et al.* Hepatocyte nuclear factor 4alpha contributes to thyroid hormone homeostasis by cooperatively regulating the type 1 iodothyronine deiodinase gene with GATA4 and Kruppel-like transcription factor 9. *Mol Cell Biol* 28, 3917-3931 (2008).

5. Inagaki T, *et al.* Obesity and metabolic syndrome in histone demethylase JHDM2a-deficient mice. *Genes Cells* 14, 991-1001 (2009).

6. Takayasu S, *et al.* A neuropeptide ligand of the G protein-coupled receptor GPR103 regulates feeding, behavioral arousal, and blood pressure in mice. *Proc Natl Acad Sci U S A* 103, 7438-7443 (2006).

7. Sakakibara I, *et al.* Fasting-induced hypothermia and reduced energy production in mice lacking acetyl-CoA synthetase 2. *Cell Metab* 9, 191-202 (2009).

8. Bachman ES, *et al.* betaAR signaling required for diet-induced thermogenesis and obesity resistance. *Science* 297, 843-845 (2002).

9. Sun L, *et al.* Mir193b-365 is essential for brown fat differentiation. *Nat Cell Biol* 13, 958-965 (2011).

10. Okamura M, *et al.* COUP-TFII acts downstream of Wnt/beta-catenin signal to silence PPARgamma gene expression and repress adipogenesis. *Proc Natl Acad Sci U S A* 106, 5819-5824 (2009).

11. Goda S, Isagawa T, Chikaoka Y, Kawamura T, Aburatani H. Control of histone H3 lysine 9 (H3K9) methylation state via cooperative two-step demethylation by Jumonji domain containing 1A (JMJD1A) homodimer. *J Biol Chem* 288, 36948-36956 (2013).
